# L3MBTL1, a polycomb protein, promotes Osimertinib acquired resistance through epigenetic regulation of DNA damage response in lung adenocarcinoma

**DOI:** 10.1038/s41419-024-06796-2

**Published:** 2024-09-04

**Authors:** Zihe Zhang, Yongwen Li, Ruifeng Shi, Chaoyi Jia, Songlin Xu, Guangsheng Zhu, Peijun Cao, Hua Huang, Xuanguang Li, Hongbing Zhang, Minghui Liu, Chen Chen, Hongyu Liu, Chunsheng Kang, Jun Chen

**Affiliations:** 1 Department of Lung Cancer Surgery, Tianjin Medical University General Hospital, Tianjin, China; 2 Tianjin Key Laboratory of Lung Cancer Metastasis and Tumor Microenvironment, Tianjin Lung Cancer Institute, Tianjin Medical University General Hospital, Tianjin, China; 3 Department of Thoracic Surgery and Oncology, the First Affiliated Hospital of Guangzhou Medical University, State Key Laboratory of Respiratory Disease, National Clinical Research Center for Respiratory Disease, Guangzhou Institute of Respiratory Health, Guangzhou, China; 4 Department of Neurosurgery, Tianjin Medical University General Hospital, Lab of Neuro-oncology, Tianjin Neurological Institute, Key Laboratory of PostNeuroinjury Neuro-repair and Regeneration in Central Nervous System, Ministry of Education and Tianjin City, Tianjin, 300052 China; 5 Department of Thoracic Surgery, First Affiliated Hospital, School of Medicine, Shihezi University, Shihezi, China

**Keywords:** Oncogenesis, Non-small-cell lung cancer

## Abstract

Osimertinib is a third-generation epidermal growth factor receptor (EGFR) tyrosine kinase inhibitor (EGFR-TKI) approved for patients with EGFR T790M resistance mutations as first- or second-line treatment of EGFR-positive patients. Resistance to Osimertinib will inevitably develop, and the underlying mechanisms are largely unknown. In this study, we discovered that acquired resistance to Osimertinib is associated with abnormal DNA damage response (DDR) in lung adenocarcinoma cells. We discovered that the polycomb protein Lethal(3) Malignant Brain Tumor-Like Protein 1 (L3MBTL1) regulates chromatin structure, thereby contributing to DDR and Osimertinib resistance. EGFR oncogene inhibition reduced L3MBTL1 ubiquitination while stabilizing its expression in Osimertinib-resistant cells. L3MBTL1 reduction and treatment with Osimertinib significantly inhibited DDR and proliferation of Osimertinib-resistant lung cancer cells in vitro and in vivo. L3MBTL1 binds throughout the genome and plays an important role in EGFR-TKI resistance. It also competes with 53BP1 for H4K20Me2 and inhibits the development of drug resistance in Osimertinib-resistant lung cancer cells in vitro and in vivo. Our findings suggest that L3MBTL1 inhibition is a novel approach to overcoming EGFR-TKI-acquired resistance.

## Introduction

Lung cancer is the world’s second most common malignant tumor in terms of morbidity and mortality, according to the most recent World Cancer Statistics Report published in 2022 [1]. Lung cancer treatment has advanced significantly over the last few decades. However, the overall survival rates are only around 15%. Epidermal growth factor receptor (EGFR) tyrosine kinase inhibitors (TKIs) have emerged as first-line therapy for patients with advanced non-small-cell lung cancer (NSCLC) harboring EGFR-TKI-sensitive mutations, because of their higher response rate and lower toxicity compared to conventional chemotherapy. However, the majority of patients develop drug resistance 4–12 months after starting treatment with the first-generation targeted drugs, Gefitinib or Erlotinib, which leads to tumor recurrence and metastasis. Resistance to first-generation EGFR-TKIs can be caused by several mechanisms, including the development of a secondary EGFR mutation (such as T790M), MET gene amplification, or activation of other pathway molecules. The third-generation EGFR-TKI Osimertinib (AZD9291), which was designed to overcome first-generation EGFR-TKI resistance, has received marketing approval [2]. Osimertinib irreversibly targets the EGFR exon 19 deletion, L858R, and T790M mutations, and is approved for patients who have the EGFR T790M resistance mutation in the first- and second-line setting for all EGFR-positive patients. Regardless of its efficacy, resistance to Osimertinib develops, and, unfortunately, resistance to Osimertinib confers resistance to all current EGFR inhibitors. Thus, it is necessary to identify the underlying mechanisms to overcome acquired Osimertinib resistance. The EGFR C797S mutation is the most common mutation associated with acquired resistance to Osimertinib, accounting for 10–26% of cases in second-line therapy and 7% in first-line treatment. The second most common mutation is MET rearrangement (15%). It involves cell cycle modifications, HER2 amplification, and activation of the PI3K pathway. Roper [3] conducted a phylogenetic tree analysis of tumor tissues following the development of Osimertinib resistance and discovered that two or more subclonal changes could occur. The underlying molecular mechanism included neuroendocrine differentiation, PD-L1 and KRAS amplification, ESR1-AKAP12, and MKRN1-BRAF fusion without histological transformation; however, the mechanisms for a wide range of cases are unknown.

Genomic DNA is important for cell homeostasis because it is the primary genetic information carrier [4]. Numerous environmental and endogenous factors continue to attack genomic DNA. The most severe effects of genomic DNA damage are facilitated by DNA double-strand breaks (DSB). Because there is no intact complementary strand to serve as a template for DNA repair, it not only results in genetic material loss but also in chromatin loss, breakage, and rearrangement, which severely impairs cell viability and promotes tumorigenesis [5]. Cells have evolved a sophisticated and complex DNA damage response (DDR) during continuous evolution and growth to deal with genomic DNA instability caused by internal and external environments. This system monitors DNA damage, repairs it, regulates the cell cycle, and changes the chromatin structure [6]. Studies have shown that altering the DDR or inhibiting the damage repair pathway contributes to drug resistance; however, it is unclear whether DNA damage reduction contributes to Osimertinib resistance.

For DNA damage signaling and repair, chromatin remodeling is most likely required. Following the occurrence of a DSB, the chromatin structure initially relaxes, which is dependent on PARP1. Chromatin relaxation is activated to help repair and checkpoint proteins enter the DNA damage site. Alternatively, the relaxation of chromatin near the damage site may result in the initial activation of ATM. Subsequently, the chromatin structure re-establishes cohesion, and this sustained chromatin compaction not only prevents DNA damage from occurring but also enhances ATM and ATR-related upstream DDR signaling [7]. Recently, a variety of chromatin remodeling mechanisms associated with the cellular response to DNA damage were described [8–10]. Lethal(3) Malignant Brain Tumor-Like Protein 1 (L3MBTL1) is located on the long arm of chromosome 20q, specifically at 20q12. It is the human homolog of Drosophila’s polycomb group PcG L(3) malignant brain tumor (MBT), which has three MBT repeats, one zinc finger, and one SPM dimer domain [11]. L3MBTL1 causes chromatin structure condensation by binding mono- and dimethylated histones H4K20 and H1bK26, resulting in a condensed nucleosome array. It is worth noting that the TUDOR domain of 53BP1 and the second MBT have very similar binding modes with histone mono- and demethylation [12]. L3MBTL1 competes with H4K20Me2 for the ability to inhibit the interaction between 53BP1 and H4K20Me2, altering the function of 53BP1 at the site of DNA damage. RNF8-mediated ATPase valosin-containing protein (VCP)/p97 promotes the ubiquitination of L3MBTL1 following the occurrence of DSB, which in turn, promotes the normal binding of 53BP1 and H4K20Me2 [13]. L3MBTL1 is also involved in various aspects of DNA damage and DNA replication [14], but its role in Osimertinib resistance has yet to be determined.

In this study, we investigated the DDR after Osimertinib treatment, as well as chromatin remodeling, with a focus on L3MBTL1-mediated condensation of chromatin and EGFR-TKI resistance.

## Results

### The DNA damage response is involved in tumor recurrence and metastasis in lung adenocarcinoma patients

**Fig. 1:**
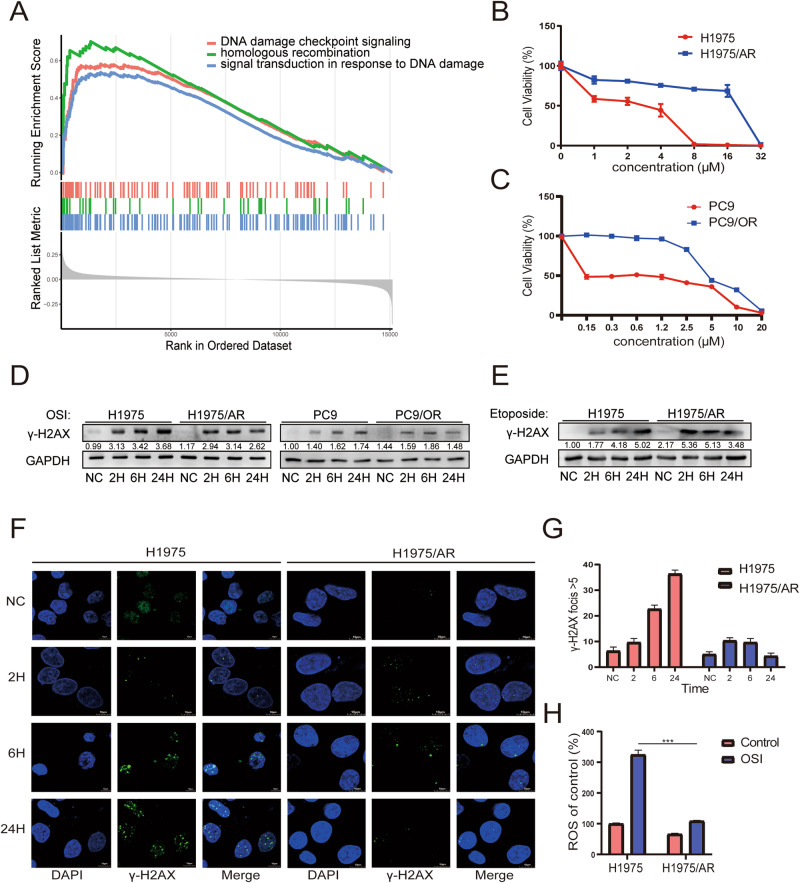
Comparison of DNA Damage Response Variations in Relapse Resistant and Sensitive Tissues and Cells. **A** DNA damage response pathway differential gene enrichment is observed in 85 lung adenocarcinoma patients screened with dataset GSE30219 using KEGG analysis. **B**, **C** CCK-8 detection of H1975 and H1975/AR, PC9, and PC9/OR cells viability after treatment with various doses of Osimertinib for 48 h. The experiments included three replicates, and the results are presented as mean ± SEM. **D** Immunoblotting was used to detect the effect of Osimertinib treatment in H1975 and H1975/AR, PC9, and PC9/OR on the expression of γ-H2AX over time. **E** Immunoblotting was used to detect the effect of Etoposide treatment in H1975 and H1975/AR, PC9, and PC9/OR on the expression of γ-H2AX over time. **F**, **G** Immunofluorescence staining of H1975 and H1975/AR cells with γ-H2AX antibody after treatment with Osimertinib over time. Count and analyze cells with γ-H2AX focis>5. Scale bar: 10 µm. The experiments included three replicates, and the results are presented as mean ± SEM. **H** H1975 and H1975/AR were treated with Osimertinib, and the level of ROS was measured using DCFH-DA staining. (mean ± SD, n = 3, *P < 0.05, **P < 0.01, ***P < 0.001).

We analyzed the GSE30219 dataset from the NCBI GEO database to identify differences in biological function and signaling pathways between primary lung adenocarcinoma tissues and recurrent tumor tissues. Whole transcriptome sequencing was performed on tumor tissues from 85 patients with lung adenocarcinoma, including 58 primary tissues and 27 tissues after tumor recurrence. Signaling pathways were annotated using KEGG pathway analysis, which revealed that differentially expressed genes (DEGs) were also enriched in DNA damage checkpoint signaling, homologous recombination, and signal transduction in response to the DDR. This suggests that post-recurrence lung adenocarcinoma tissue has a high correlation with the DDR (Fig. 1A, Fig. S1A). Using these results, we thereby investigated whether there was a link between DDR and tumor recurrence caused by resistance to targeted therapy in lung adenocarcinoma.

### EGFR signaling inhibition activates the DNA damage response in Osimertinib-resistant lung adenocarcinoma

To determine whether the DDR plays a role in the development of acquired resistance of lung adenocarcinoma cells to Osimertinib, we exposed the lung adenocarcinoma cell lines, NCI-H1975 and PC9, to Osimertinib. NCI-H1975 is a lung adenocarcinoma cell line with L858R and T790M EGFR mutations that are sensitive to Osimertinib, whereas PC9 cells have a Del19 EGFR mutation and are also sensitive to Osimertinib. Following prolonged stimulation with Osimertinib, the half-maximal inhibitory concentration *(IC50)* value of Osimertinib was determined using a CCK-8 assay. The IC50 of the H1975 cell line was 1.78 μmol/L, while the IC50 of the H1975/AR (stands for H1975 AZD9291 resistant) subline was 12.3 μmol/L (Fig. 1B). The IC50 for the PC9 cell line was 0.66 μmol/L, while the IC50 of the PC9/OR (stands for Osimertinib resistant) subline was 5.40 μmol/L (Fig. 1C), indicating that Osimertinib acquired-resistant cell lines were established. Meanwhile, we validated the in vivo results by subcutaneously growing H1975 and H1975/AR cell lines in nude mice. When the tumor volume exceeded 200 mm^2^ the tumors were treated with Osimertinib via gavage. Compared with the decreasing size of H1975 tumors, H1975/AR tumors continued to grow, indicating that they were resistant to Osimertinib in vivo (Fig. S1B). After treating H1975 and H1975/AR cells with the same concentration of Osimertinib for different durations, immunoblotting revealed that EGFR and its downstream pathway proteins(Fig. S1C), such as p-AKT and p-MAPK expression, were inhibited in H1975 and H1975/AR cells. In contrast, the expression of γ-H2AX in H1975 and PC9 cells increased. However, in H1975/AR and PC9/OR cells, the expression of γ-H2AX briefly increased after drug treatment but was reduced after 24 h (Fig. 1D). γ-H2AX, a phosphorylated histone protein, acts as a marker for DNA DSBs [15]. To confirm the link between decreased DNA damage and acquired resistance to Osimertinib, we used Etoposide, a chemical that inhibits DNA synthesis and induces double-stranded DNA breaks by forming a complex with Topoisomerase II and DNA [16]. We observed that γ-H2AX remained consistently elevated in H1975 and decreased after 24 h with Osimertinib in H1975/AR(Fig. 1E). A similar result was obtained using immunofluorescence to observe and count γ-H2AX-foci (Fig. 1F–G). A Comet assay was then used to confirm the DNA damage. DNA damage was evident after 24 h of Osimertinib treatment in H1975 cells, whereas in H1975/AR cells, DNA damage increased within 6 h and decreased after Osimertinib treatment for 24 h. H1975 cells sustained more DNA damage than H1975/AR during the same period (Fig. S1D, E). To determine the mechanism of DNA damage caused by Osimertinib and the differences in the degree of DNA damage between H1975 and H1975/AR cells, we hypothesized that changes in reactive oxygen species (ROS) levels could be involved in DNA damage caused by Osimertinib. Elevated ROS levels cause oxidative stress and damage to proteins, lipids, and DNA [17]. ROS levels in H1975 cells are higher than those in H1975/AR with growth medium (p < 0.001). Osimertinib treatment increased ROS levels in both H1975 and H1975/AR, but H1975 had higher levels than H1975/AR (p < 0.001). This suggests that the increase in ROS after Osimertinib treatment is lower in resistant cells than in sensitive cells (Fig. 1H), making these cells more resistant to oxidative stress and reducing DNA damage.

**Fig. 2:**
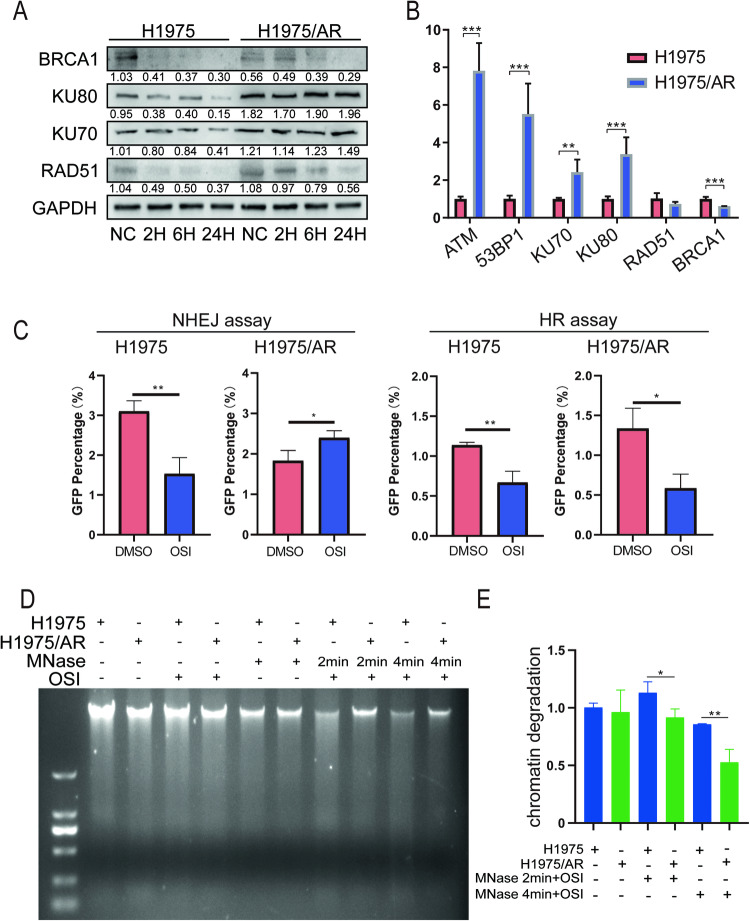
A comparison of the differences between acquired resistant and sensitive cells’ chromatin structures and DNA repair pathways. **A** Immunoblotting was used to determine the effect of Osimertinib treatment in H1975 and H1975/AR on the expression of key NHEJ and HR proteins over time. **B** qRT-PCR measured mRNA levels of ATM, 53BP1, KU70, KU80, RAD51 and BRCA1 between H1975 and H1975/AR (mean ± SD, n = 3, *P < 0.05, **P < 0.01, ***P < 0.001). **C** NHEJ efficiency and HR efficiency were determined by quantifying GFP expression (by flow cytometry) in H1975 and H1975/AR cells harboring pDR-GFP reporter or pimEJ5GFP after transfection with a pCBASceI plasmid with or without treated Osimertinib for 48 h. (mean ± SD, n = 3, *P < 0.05, **P < 0.01, ***P < 0.001). **D**, **E** Micrococcal Nuclease digestion assay determined the extent of MNase sensitivity in H1975 and H1975/AR cells with Osimertinib treated.

In eukaryotic organisms, DSBs repair DNA damage using two mechanisms: Non-homologous end joining (NHEJ) and homologous recombination (HR) [18, 19].To determine the variations in these repair pathways in the two pairs of cell lines after Osimertinib treatment, we measured KU70 and KU80, which are involved in the NHEJ pathway, and RAD51 and BRCA1, which are involved in the HR pathway, using immunoblotting. In H1975 cells, Osimertinib reduced the expression of genes involved in the HR and NHEJ repair pathways. BRCA1 and RAD51 expression decreased in H1975/AR, while KU70 and KU80 expression increased (Fig. 2A). We also saw a similar pattern in PC9 and PC9/OR cell lines (Fig. S1F). We used qRT-PCR to measure the expression of the aforementioned DNA repair genes in the two cell lines and discovered that the NHEJ pathway genes, KU70 and KU80, were highly expressed in H1975/AR, whereas the HR repair pathway genes, RAD51 and BRCA1, were either unchanged or decreased in expression. Furthermore, we discovered that the expression of ATM, an important upstream gene for DNA damage repair, was also increased (Fig. 2B). An NHEJ/HR assay was performed to confirm the findings presented above. Compared with the DNA damage group caused by I-SceI, Osimertinib treatment inhibited the NHEJ and HR pathways in H1975 cells (p < 0.01 and p < 0.01), while in H1975/AR cells, the HR pathway was inhibited but the NHEJ pathway was abnormally increased (p < 0.05 and p < 0.05) (Fig. 2C). The results were consistent with immunoblotting, which revealed high expression of the upstream DNA damage repair signal in H1975/AR. The findings suggest that one of the causes of acquired resistance to Osimertinib could be an abnormal increase in the NHEJ repair pathway.

Chromatin relaxation and condensation are the initial effects of DSBs, which are required for both DSB damage and repair [7]. Therefore, we investigated whether chromatin structure changes contribute to EGFR-TKI resistance. Chromatin relaxation analysis consistently showed that chromatin degradation in H1975 cells was more evident than in H1975/AR cells following the addition of micrococcal nuclease (MNase) after Osimertinib treatment (Fig. 2D, E). These findings show that the chromatin of H1975/AR cells is more condensed than that of H1975 cells after Osimertinib treatment, implying that cells may increase their resistance to oxidative stress to reduce DNA damage. Furthermore, chromatin condensation may enhance the ATM-related upstream DDR signal.

### EGFR oncogene inhibition reduces L3MBTL1 ubiquitination and stabilizes its expression in Osimertinib-resistant cells

**Fig. 3:**
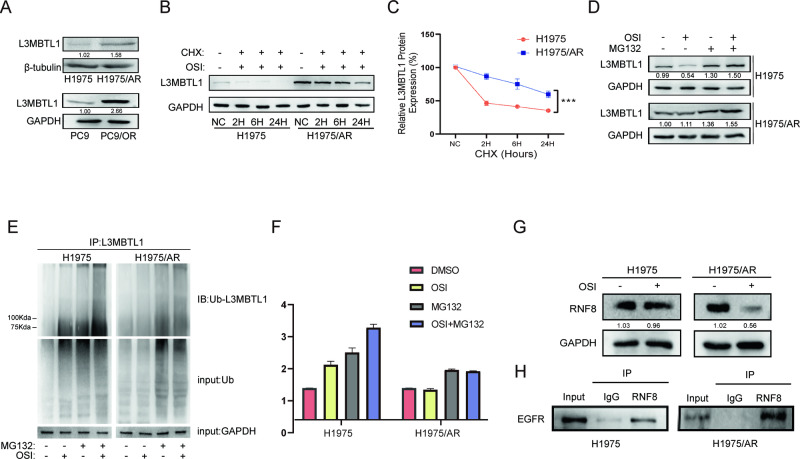
Osimertinib-resistant cells showed more stable L3MBTL1 expression at the protein level. **A** Immunoblotting was used to measure the protein level of L3MBTL1 in H1975 and H1975/AR, as well as PC9 and PC9/OR. **B**, **C** H1975 and H1975/AR were treated with Osimertinib and cycloheximide (CHX) alone or in combination over time. L3MBTL1 protein expression was detected by immunoblotting and relative density was assessed using Image J (mean ± SD, n = 3, *P < 0.05, **P < 0.01, ***P < 0.001). **D** H1975 and H1975/AR cells were treated with Osimertinib and MG132 separately or together over time. L3MBTL1 protein expression was detected by immunoblotting, and relative density was measured using Image J (mean ± SD, n = 3, *P < 0.05, **P < 0.01, ***P < 0.001). **E**, **F** H1975/AR has a lower ubiquitin level than the corresponding H1975, MG132 combination for rescue. After 48 h of treatment with 2 μM Osimertinib, cell lysates were immunoprecipitated with rabbit anti-L3MBTL1 antibody, followed by anti-ubiquitin antibody for immunoblotting, and relative density was measured using Image J (mean ± SD, n = 3, *P < 0.05, **P < 0.01, ***P < 0.001). **G** Immunoblotting was used to assess the effect of Osimertinib treatment in H1975 and H1975/AR on the expression of RNF8. **H** The co-immunoprecipitate assay was used to determine the binding of EGFR to RNF8 in H1975 and H1975/AR.

MBT domain proteins play a role in the condensation of chromatin structure, but their relationship to acquired Osimertinib resistance is unclear. We measured the MBT domain family member L3MBTL1 to determine whether the expression of MBT domain proteins changed in Osimertinib acquired-resistant cells. The results indicated that L3MBTL1 protein was highly expressed in H1975/AR cells (P < 0.001), as well as in PC9/OR cells, which had higher expression than PC9 cells (Fig. 3A). To better understand the reason for the higher expression of L3MBTL1 in drug-resistant cells compared to sensitive cells, we tracked the dynamics of L3MBTL1 protein expression in the two cell types after Osimertinib stimulation over time. Following Osimertinib treatment, we observed that L3MBTL1 gradually decreased in H1975 cells but remained elevated for at least 72 h in H1975/AR cells (Fig. S2A). To investigate the molecular regulation of L3MBTL1 proteins with different equilibria in these two cell types, we performed protein stability measurements of L3MBTL1 using cycloheximide, an intracellular protein synthesis inhibitor and found that the half-life of L3MBTL1 in H1975/AR cells increased after treatment with the cycloheximide combined with Osimertinib, indicating that the L3MBTL1 protein in H1975/AR cells is more stabilized than in H1975 (Fig. 3B-C) after DNA strand breaks [13]. We then used MG132, a proteasome inhibitor, and discovered that L3MBTL1 increased after co-treatment with Osimertinib (Fig. 3D). We also investigated whether ubiquitination affects the stability of the L3MBTL1 protein and quantified L3MBTL1 ubiquitination levels in H1975 and H1975/AR cells. The results indicated that the ubiquitination of L3MBTL1 increased significantly in H1975 cells but decreased slightly lower in H1975/AR cells following Osimertinib treatment. MG132 inhibited L3MBTL1 ubiquitination degradation when combined with Osimertinib (Fig. 3E, F). As a result of Osimertinib treatment, the ubiquitination level of the L3MBTL1 protein in H1975/AR cells decreased, resulting in greater protein stability compared to H1975 and higher expression of L3MBTL1.

As an E3 ubiquitin ligase, RNF8 increase the ubiquitination of L3MBTL1 by promoted the recruitment of ATPase VCP/p97 to DNA damage sites after DSB [13]. Immunoblotting showed that Osimertinib significantly reduced RNF8 expression in H1975/AR cells (Fig. 3G). We also performed immunoprecipitation assays using anti-RNF8 and discovered that RNF8 co-precipitates with EGFR in H1975 and H1975/AR cells (Fig. 3H). Accordingly, we deduced that in H1975/AR cells, a decrease in RNF8 expression also mediates the abnormal up-regulation of L3MBTL1, resulting in elevated L3MBTL1 expression in EGFR-TKI acquired-resistant cells.

### L3MBTL1 is involved in the DNA damage process by regulating chromatin remodeling

**Fig. 4:**
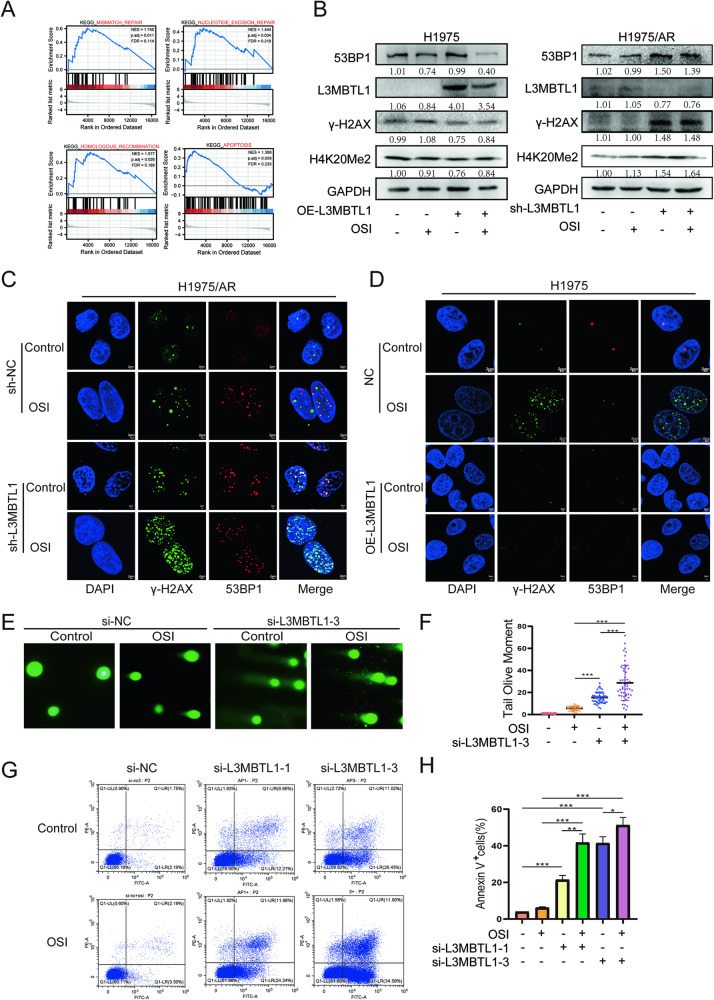
L3MBTL1 compacts the chromatin structure, reducing cellular DNA damage. **A** The enrichment of signaling pathways among differentially expressed genes was investigated using gene set enrichment analysis (GSEA). DEGs were discovered to be enriched in DDR-related pathways such as apoptosis, homologous recombination, mismatch repair, and nucleotide excision repair. **B** H1975 and H1975/AR were transfected into OE-L3MBTL1 and sh-L3MBTL1, respectively, and treated with or without Osimertinib. 53BP1, L3MBTL1, γ-H2AX, and H4K20Me2 protein levels were evaluated by immunoblotting. **C** H1975/AR sh-NC and H1975/AR sh-L3MBTL1 were treated with Osimertinib or DMSO, γ-H2AX and 53BP1 focis can be observed by immunofluorescence staining. Scale bar: 2 µm. **D** H1975 OE-NC and H1975 OE-L3MBTL1 were treated with Osimertinib or DMSO, γ-H2AX and 53BP1 focis can be observed by immunofluorescence staining. Scale bar: 2 µm. **E**, **F** H1975/AR si-NC and H1975/AR si-L3MBTL1-3 were treated with DMSO or Osimertinib, DNA damage was measured using the Comet assay, and the tail olive moment was analyzed using Cometscore (mean ± SD, n = 3, *P < 0.05, **P < 0.01, ***P < 0.001). **G**, **H** After H1975/AR was transfected into si-NC, si-L3MBTL1-1 and si-L3MBTL1-3 for 48 h, DMSO or Osimertinib were treated, respectively, and the apoptosis level was detected by flow cytometry (mean ± SD, n = 3, *P < 0.05, **P < 0.01, ***P < 0.001).

Following L3MBTL1 knockdown in H1975/AR cells, we generated gene expression profiles to determine the underlying mechanism. RNA-seq was used to distinguish DEGs between H1975/AR sh-NC and H1975/AR sh-L3MBTL1 cells. To avoid false positives, three control experiments were run concurrently, with a threshold minimum of a 2*-*fold difference serving as the exclusion criteria. We identified 1663 DEGs, with 748 increasing and 915 decreasing (Fig. S2B). Gene Set Enrichment Analysis (GSEA) was used to examine functional enrichment. Apoptosis, HR, mismatch repair, and nucleotide excision repair are among the DDR mechanisms linked to altered gene expression (Fig. 4A). Therefore, we hypothesized that L3MBTL1 mediates Osimertinib resistance via the DDR. We used siRNA to reduce the expression of L3MBTL1. The efficiency of mRNA knockdown is depicted in Fig. S2C, with si-L3MBTL1-1 and si-L3MBTL1-3 selected to reduce the possibility of off-target effects. In H1975/AR cells, the expression of γ-H2AX increased after knocking down L3MBTL1, as well as 53BP1, H4K20Me2. Subsequently, we overexpressed L3MBTL1 in H1975 cells and found that the expression of γ-H2AX, 53BP1, and H4K20Me2, were all decreased (Fig. 4B). The results were confirmed by immunofluorescence, and the result revealed that compared to the control group, the foci formed by γ-H2AX and 53BP1 in H1975 cells overexpressing L3MBTL1 had significantly decreased. However, the formation of foci increased significantly after L3MBTL1 knockdown in H1975/AR cells and became more visible after treatment with Osimertinib (Fig. 4C, D). We also confirmed these changes in PC9/OR using immunoblotting (Fig. S2D). These findings were further confirmed in a Comet assay of H1975/AR cells, in which the tail length increased following L3MBTL1 knockdown (0.9325 vs. 15.44, p < 0.001), while the tail length was further increased after L3MBTL1 knockdown and treatment with Osimertinib (15.44 vs. 28.70, p < 0.001) (Fig. 4E, F). Since ROS is involved in H1975/AR for the extent of DNA damage, we discovered that knockdown of L3MBTL1 combined with Osimertinib treatment increased ROS levels (Fig. S2E), resulting in increased DNA damage. The NHEJ pathway was abnormally elevated in H1975/AR, we discovered that the knockdown of L3MBTL1 combined with Osimertinib inhibited the NHEJ pathway, preventing cellular repair (Fig. S2F). We also investigated the stability of the L3MBTL1 protein and discovered that the protein half-life decreased more rapidly after the knockdown of L3MBTL1 than in the control group (Fig. S2G, H). In addition, we discovered that si-L3MBTL1 in combination with Osimertinib caused apoptosis in H1975/AR cells as shown in Fig. 4G, H. An analysis of Annexin V-FITC/PI double-staining in H1975/AR cells revealed that apoptosis and necrotic cells increased after L3MBTL1 knockdown (p < 0.001 and p < 0.05). These findings show that L3MBTL1 promotes DNA damage, and when combined with Osimertinib, DNA damage, genomic instability, and apoptosis all increased.

The accumulation of DNA damage may be affected by altered chromatin and chromosome structures. To determine whether L3MBTL1 is responsible for the abnormal compaction of chromatin structures in Osimertinib-resistant cells, Osimertinib was given to H1975/AR sh-L3MBTL1 cells for 24 h. A micrococcal nuclease digestion assay was then performed on both cell lines to determine whether variations in L3MBTL1 expression changed chromatin conformation. Larger diffuse DNA fragments were found in the chromatin of H1975/AR sh-L3MBTL1 cells. The DNA fragments were more degraded than in the control group, and after Osimertinib treatment for 24 h, the chromatin structure was looser (Fig. S2I, J). These findings suggest that L3MBTL1 reduces the accumulation of DNA damage during Osimertinib treatment while increasing resistance by condensing chromatin structures in H1975/AR cells.

### L3MBTL1 binds throughout the genome and plays a critical role in EGFR-TKI resistance

**Fig. 5:**
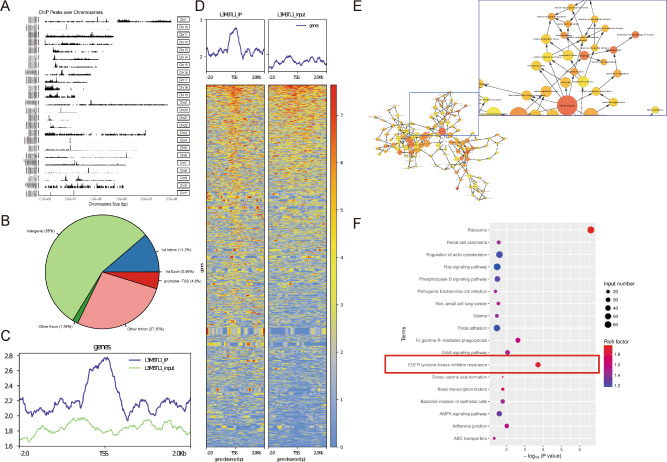
L3MBTL1 plays an important role in EGFR-TKI resistance. **A** Distribution of L3MBTL1 Peaks in Chromatin. **B**, **C** The distribution of peaks in the functional regions of the genome accounted for 4.8% of the peaks in Promoter-TSS. **D** Distribution and heatmap of Peaks above and below TSS. **E** A GO analysis of genes related to L3MBTL1 promoter peaks revealed enrichment in cellular processes such as cell cycle, cell metabolism, and cellular stress response. **F** A KEGG analysis revealed that L3MBTL1 promoter peaks-related genes were highly enriched in EGFR and its downstream pathways, as well as some EGFR-TKI-related bypass pathways.

L3MBTL1 is classified as a chromatin compaction factor [11]. We used whole-genome ChIP-seq in H1975/AR cells to determine the distribution of L3MBTL1 across the genome. The findings revealed the genome-wide distribution of L3MBTL1 (Fig. 5A). A total of 19,565 L3MBTL1 promoter peaks were counted, accounting for 4.8% of the transcription initiation points (TSS) using input as the background and Epic2 to call the IP peaks (Fig. 5B–D). The peaks were subjected to gene association analysis, which revealed 4782 peak-related genes. A GO analysis revealed that the genes were enriched in cellular processes (e.g., cell cycle, cell metabolism, and cell stress response) and cell signal transduction (Fig. 5E). A KEGG analysis revealed that genes associated with the L3MBTL1 promoter peaks were highly enriched in EGFR and downstream signaling pathways, as well as some EGFR-TKI-related bypass pathways (Fig. 5F). These findings indicate that L3MBTL1 plays an important role in the development of EGFR-TKI resistance in NSCLC cells.

### Effect of L3MBTL1 on the sensitivity of resistant lung cancer cells to Osimertinib in vitro

**Fig. 6:**
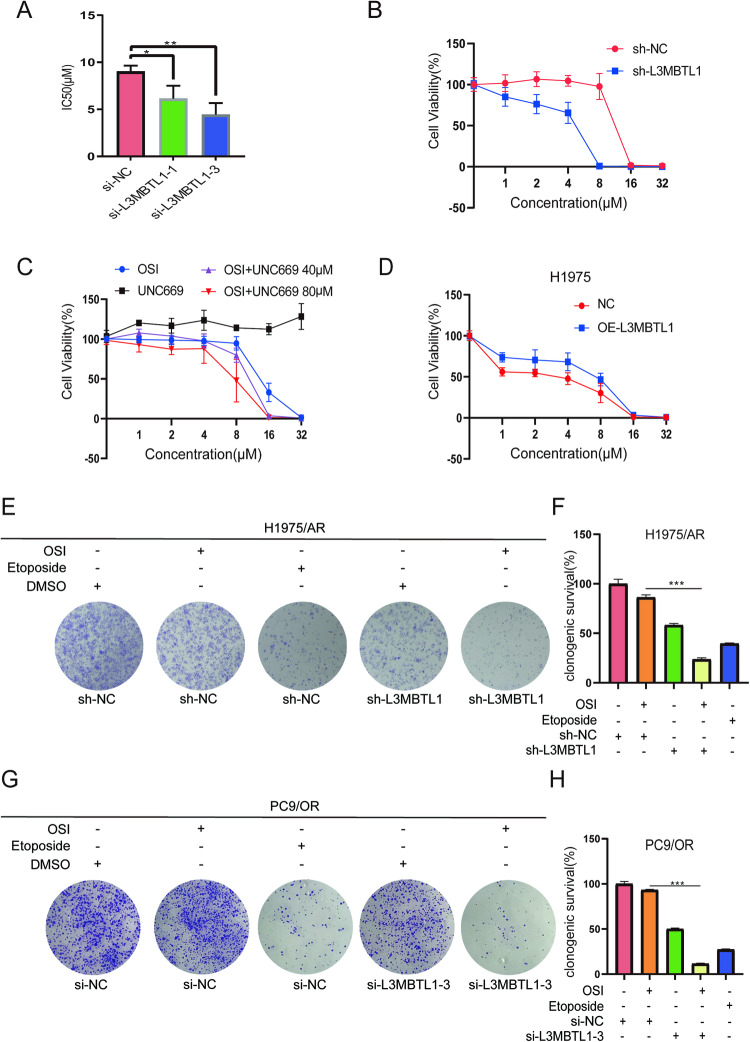
L3MBTL1 inhibition enhances the antitumor effect of EGFR-TKIs in vitro. After cells transfected with si-NC, si-L3MBTL1-1, or si-L3MBTL1-3 48 h as indicated, with treated for DMSO or Osimertinib, IC50 were determined using the CCK-8 assay (**A**) in H1975/AR. (mean ± SD, n = 3, *P < 0.05, **P < 0.01, ***P < 0.001). **B** After transfecting H1975/AR into mock lentiviral or sh-L3MBTL1, CCK-8 was used to detect cell viability after various doses of Osimertinib were treated for 48 h. The experiments included three replicates, and the results are presented as mean ± SEM. **C** Cell viability of H1975/AR was assessed using the CCK-8 assay when treated with Osimertinib and UNC669 alone and in combination. The experiments included three replicates, and the results are presented as mean ± SEM. **D** After transfecting H1975 into mock lentiviral or OE-L3MBTL1, CCK-8 was used to detect cell viability after various doses of Osimertinib were treated for 48 h. The experiments included three replicates, and the results are presented as mean ± SEM. **E**–**H** Cell proliferation was assessed using clonogenic assays in H1975/AR and PC9/OR cells after transfection with empty vector or L3MBTL1 knockdown for the indicated number of hours and treated with DMSO or Osimertinib, Etoposide for positive control (mean ± SD, n = 3, *P < 0.05, **P < 0.01, ***P < 0.001).

To evaluate the effect of L3MBTL1 knockdown on drug sensitivity in H1975/AR cells, we calculated the *IC50* values for cells treated with Osimertinib using a CCK*-*8 assay. After the knockdown of L3MBTL1 with si-L3MBTL1-1 and si-L3MBTL1-3, the *IC50* values decreased by 31.9% and 50.9%, respectively, compared to the control group (p < 0.05 and p < 0.01) (Fig. 6A). We performed a similar test on H1975/AR cells following L3MBTL1 knockdown with lentivirus. The *IC50* value in the L3MBTL1 shRNA knockdown group decreased by 72.3% when compared to the control group (Fig. 6B), which supports our previous findings. We also investigated the efficacy of a potent and selective MBT inhibitor, UNC669, which shows greater selectivity for L3MBTL1 than other MBT family homologs. When administered alone, UNC669 did not affect cell viability. However, when combined with Osimertinib at a fixed concentration, the inhibition rate exceeded 95% at an Osimertinib concentration of 16 µM (compared to 67% for Osimertinib alone). The Combination index was 0.4 calculated by the CompuSyn, indicating a significant synergistic effect (Fig. 6C), implying that UNC669 in combination with Osimertinib may have a synergistic effect on inhibiting cell growth. In contrast, the *IC50* value increased by 103.8% in H1975 cells overexpressing L3MBTL1, as revealed by CCK-8 analysis, indicating resistance to Osimertinib (Fig. 6D). Furthermore, L3MBTL1 knockdown significantly increased the sensitivity of PC9/OR cells to Osimertinib, lowering the IC_50_ value by 44.2% (Fig. S2K).

To investigate whether L3MBTL1 expression works synergistically with Osimertinib in H1975/AR and PC9/OR cells, we divided the cells into five groups: scrambled, Osimertinib single-drug treatment, L3MBTL1 knockdown, Etoposide group, and L3MBTL1 knockdown plus Osimertinib treatment. Colony formation assays revealed that cell proliferation was reduced following L3MBTL1 knockdown. Moreover, there was a significant reduction in cell proliferation when L3MBTL1 knockdown was combined with Osimertinib treatment (p < 0.001) (Fig. 6E, F), as well as in the L3MBTL1-3 knockdown group containing PC9/OR cells (Fig. 6G, H). These findings suggest that inhibiting L3MBTL1 in combination with Osimertinib treatment significantly reduces the proliferation capacity of H1975/AR cells. Taken together, our findings support the hypothesis that L3MBTL1 knockdown in NSCLC cells improves Osimertinib sensitivity while L3MBTL1 overexpression confers resistance to Osimertinib.

### L3MBTL1 regulates chromatin structure by combining with histone modification in the development of drug resistance

**Fig. 7:**
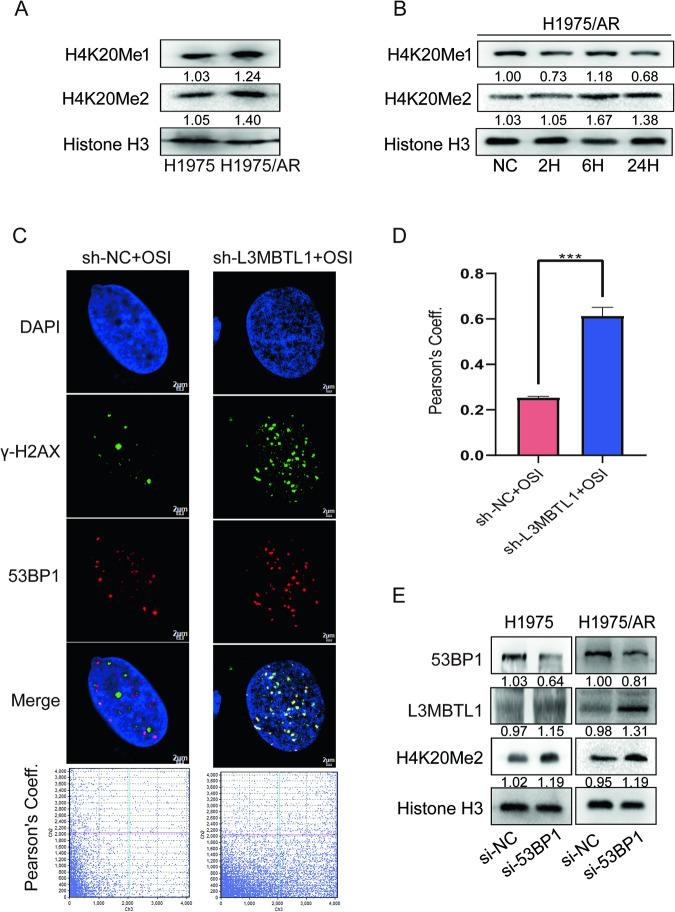
L3MBTL1 primarily competes with 53BP1 for binding to H4K20Me2 in H1975/AR cells. Immunoblotting revealed protein levels of H4K20Me1 and H4K20Me2 in nuclear proteins of H1975 and H1975/AR (**A**), as well as changes over time in H1975/AR (**B**). **C**, **D** After treating H1975/AR sh-NC and H1975/AR sh-L3MBTL1 with Osimertinib, immunofluorescence staining revealed γ-H2AX and 53BP1 foci. Scale bar: 2 µm. Pearson’s coefficient was assessed by FluoView10-ASW 4.2. (mean ± SD, n = 3, *P < 0.05, **P < 0.01, ***P < 0.001). **E** H1975 and H1975/AR were transfected into si-NC or si-53BP1, 53BP1, L3MBTL1, and H4K20Me2, and nuclear protein levels were determined using immunoblotting.

L3MBTL1 has previously been shown to maintain chromatin condensation by binding mono- and dimethylated histone H4K20 and compact nucleosome arrays [11]. We discovered that H1975/AR cells expressed more H4K20Me1 and H4K20Me2 than H1975 cells (Fig. 7A). Nonetheless after treatment with Osimertinib for 24 h, the expression of H4K20Me1 did not change significantly, whereas the expression of H4K20Me2 gradually increased (Fig. 7B), which was consistent with the trend observed with L3MBTL1. Therefore, we hypothesized that H4K20Me2 works in tandem with L3MBTL1 to promote chromatin condensation in drug-resistant cells. We examined 53BP1, a DDR protein that competes with L3MBTL1 for binding to H4K20Me2 [13]. When cells were cultured with growth medium, 53BP1 cannot bind to H4K20Me2 because L3MBTL1 competitively binds to it via a unique MBT domain. Following DNA damage, L3MBTL1 is ubiquitinated, revealing the spatial location of H4K20Me2. This makes 53BP1 more likely to bind to H4K20Me2 and be recruited to the site of DNA damage, as well as other repair-related proteins to facilitate repair. For a variety of reasons, 53BP1 may be unable to accumulate at the site of damage, preventing it from recruiting downstream proteins for DNA repair [13]. To determine whether 53BP1 typically accumulates at DNA damage sites, we used an immunofluorescence assay. Fluorescence microscopy revealed foci of 53BP1 and γ-H2AX, indicating sites of DNA damage after 24 h of Osimertinib treatment. The Pearson correlation coefficient was 0.25. Figure 7C shows that some of the 53BP1 foci did not aggregate to the DNA damage site (γ-H2AX) and protein overlap was decreased. We wondered if abnormal L3MBTL1 expression and abnormal binding of L3MBTL1 to H4K20Me2 would affect the aggregation of 53BP1. Using the same treatment on H1975/AR sh-L3MBTL1 cells, we observed increased localization of 53BP1 and γ-H2AX proteins, with a Pearson correlation coefficient of 0.61 (p < 0.001) (Fig. 7C). This suggests that the proportion of 53BP1 accumulating at sites of DNA damage has increased, and L3MBTL1 is an important factor influencing 53BP1 accumulation at DNA damage sites. We used siRNA to inhibit the expression of 53BP1 in H1975 and H1975/AR cells to further confirm the relationship between L3MBTL1 and H4K20Me2 and discovered that L3MBTL1 and H4K20Me2 expression levels increased (Fig. 7E). Taken together, L3MBTL1 works with H4K20Me2 to mediate the conformational changes in chromatin after Osimertinib treatment in H1975/AR cells.

### Effect of L3MBTL1 on tumor growth and drug resistance in vivo

**Fig. 8:**
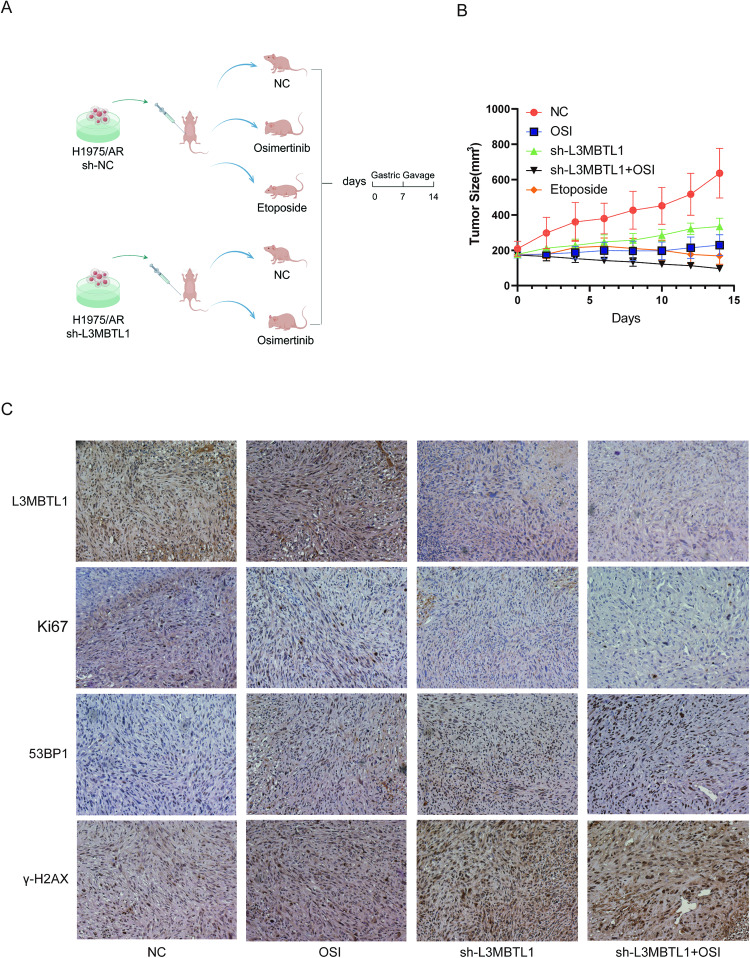
L3MBTL1 inhibition enhances the antitumor effect and DNA damage of EGFR-TKIs in vivo. **A** Schematic diagram depicting tumor formation and drug treatment time in nude mice. The design was created by Figdraw. **B** Tumor volume was measured following 2 weeks of Osimertinib treatment. Results are presented as mean ± standard deviation. **C** Immunohistochemistry was used to identify L3MBTL1, Ki67, 53BP1, and γ-H2AX in tumor sections of nude mice.

Tumor xenograft models of H1975/AR sh-NC and H1975/AR sh-L3MBTL1 cells were established in nude mice by subcutaneously injecting the cells into the animals’ right flanks to investigate the role of L3MBTL1 in Osimertinib resistance in vivo. When the tumor volume reached 150–200 mm^3^, mice representing each cell type were treated with double-distilled water or Osimertinib (2.5 mg/kg/day) by gavage for two weeks, with H1975/AR sh-NC mice receiving Etoposide as a positive control. (Fig. 8A). As shown in Fig. 8B, tumor growth rate and volume of the L3MBTL1 knockdown group were significantly lower than the control group. The combined Osimertinib treatment group demonstrated a stronger inhibitory effect. In paraffin‐embedded tissue sections, the expression of Ki-67 in tumor tissues of the L3MBTL1 knockdown plus Osimertinib group was significantly lower compared to other groups, while the expression of γ-H2AX and 53BP1 increased (Fig. 8C). The findings suggest that L3MBTL1 knockdown inhibits tumor growth while increasing DNA damage.

## Discussion

Multiple biological mechanisms of acquired resistance to Osimertinib have been identified, including secondary resistance mutations, with EGFR C797S being the most common mutation. Alternative signaling pathways, such as MET amplification, may also be activated, as are other mechanisms such as HER2 amplification, PIK3CA, KRAS, BRAF, and small cell lung cancer transformation. In general, Osimertinib resistance is complex, and the resistance mechanism for a wide range of cases is unknown. In the present study, we discovered that altered DDR, attenuated DNA damage repair, and condensed chromatin structure all contribute to Osimertinib resistance. We also discovered that the polycomb protein L3MBTL1 influences Osimertinib resistance by regulating chromatin structure and DNA damage response. EGFR oncogene inhibition decreased L3MBTL1 ubiquitination and stabilized L3MBTL1 expression, reducing DNA damage and increasing repair capacity in Osimertinib-resistant cells.

Chemotherapeutic agents target rapidly dividing cancer cells by directly or indirectly causing DNA damage. Previous research has found that altered DNA damage repair causes resistance to chemotherapy and targeted therapy drugs. For example, platinum chemotherapeutic agents, such as cisplatin and carboplatin, bind to DNA and form DNA adducts. This causes intrastrand and interstrand crosslinks, which disrupt the structure of the DNA molecule, resulting in steric changes in the helix. Alterations in the structure of the DNA molecule cause DNA damage recognition and repair, which can result in tumor cell survival and, eventually, platinum resistance. Tumor cells can have intrinsic differences in DNA repair mechanisms compared to normal cells, but these differences can also be acquired. XPA and ERCC1 in nucleotide excision repair (NER) [20], MLH1 and MLH2 in mismatch repair (MMR) [21], BRCA1 and BRCA2 in homologous recombination (HR) [22], are some examples of abnormal protein expression related to cisplatin-resistant cells and have all been linked to cisplatin resistance. The expression of the aforementioned genes may be altered to restore the sensitivity of cancer cells to cisplatin. In recent years, the link between EGFR inhibitors and DDR has been investigated. Transcription profiling of cetuximab-resistant human colorectal cancer cell lines revealed that the expression of the MMR pathway-related genes, MLH1 and MSH2, as well as the HR pathway-related genes, BRCA2 and RAD51, was decreased. The expression of the NHEJ pathway-related gene 53BP1 was increased, and an NHEJ/HR plasmid reporter assay revealed a significant decrease in HR capacity while increasing NHEJ capacity. The ROS scavenger NAC was found to reduce DNA damage, implying that cetuximab causes DNA damage via ROS accumulation. It was also demonstrated that ROS levels and DNA damage increase following exposure of cells to cetuximab [23]. According to our findings, the EGFR-TKI Osimertinib promotes oxidative stress-induced DNA damage in sensitive cells while simultaneously reducing DNA damage caused by oxidative stress tolerance in drug-resistant cells. Drug-resistant cells express more NHEJ pathway-related gene proteins than HR pathway-related gene proteins, with the latter showing decreased expression. By examining chromatin conformation, we discovered that drug-resistant cell lines have more compact chromatin than drug-sensitive cell lines. This may make cells more resistant to oxidative stress, reducing DNA damage. Therefore, we conclude that abnormal DDR is associated with acquired resistance to Osimertinib.

Some malignant tumors show abnormal expression of L3MBTL family members. Single- and double-strand deletions of L3MBTL2 and L3MBTL3 were found in medulloblastoma [24] whereas L3MBTL4 has mutations or deletions in breast cancer [25]. High L3MBTL1 expression has been linked to low-grade, hormone receptor-positive breast cancer [26]. Our findings showed that Osimertinib acquired-resistant NSCLC cells express higher levels of L3MBTL1 than sensitive cells. The decrease in RNF8 expression resulted in reduced ubiquitination of L3MBTL1 following Osimertinib treatment, allowing the expression of L3MBTL1 protein to remain stable in Osimertinib-resistant NSCLC cells. We hypothesized that L3MBTL1 primarily compacts chromatin by combining with H4K20Me2 in EGFR-TKI-acquired drug-resistant lung cancer cells, because 53BP1 did not bind to H4K20Me2, reducing DNA damage after Osimertinib treatment of Osimertinib-resistant cells.

To investigate the function of L3MBTL1 in normal and diseased conditions, Gurvich et al. [14] used the U2OS cell line and discovered that cell proliferation was inhibited following L3MBTL1 inhibition. Cell cycle analysis revealed that cells were simultaneously blocked in the G2/M phase, implying that L3MBTL1 inhibits cell growth. In contrast, Qin et al. [27] investigated L3MBTL1^−/−^ mice and discovered that L3MBTL1 did not affect normal development, reproduction, or organ development. Because no cell cycle arrest or a decrease in cyclin was observed, the researchers concluded that L3MBTL1 does not affect the growth of healthy organs and tissues. The expression of c-myc was increased in HeLa tumor cells but remained unchanged in normal mice after L3MBTL1 knockdown. This suggests that changes in L3MBTL1 do not affect the growth and development of normal tissues. We discovered that L3MBTL1 knockdown increased apoptosis. A synergistic effect was observed when Osimertinib treatment was combined with L3MBTL1 knockdown. The sensitivity of tumor cells to Osimertinib was increased by reduced L3MBTL1 expression, whereas L3MBTL1 overexpression increased the resistance. Furthermore, we discovered that L3MBTL1 and Osimertinib administered together had stronger tumor-suppressing effects in vivo.

**Fig. 9:**
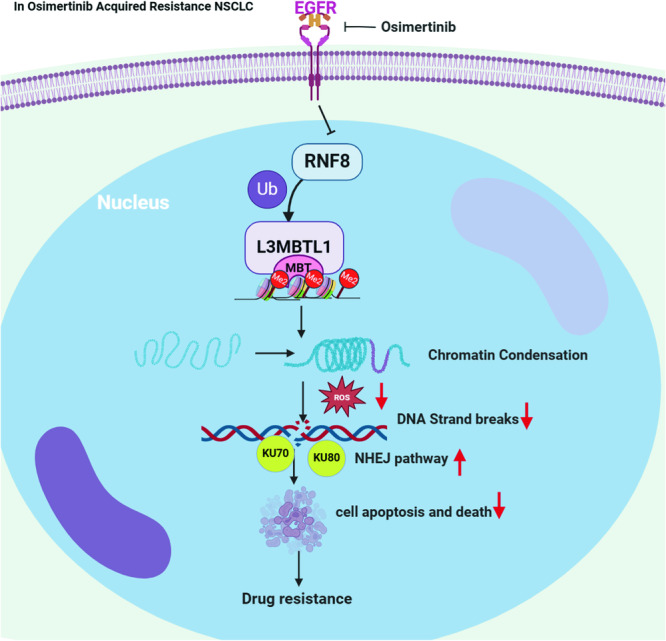
A working model for the role of L3MBTL1 acting on DNA damage response in EGFR-TKI acquired-resistant cells. In Osimertinib-resistant NSCLC, the inhibition of Osimertinib leads to a decrease in the level of L3MBTL1 ubiquitination, thereby making the chromatin more condensed, resulting in a decrease in ROS and DNA damage levels, and an increase in DNA damage repair, which increases the tumor's drug resistance. The working model is created with BioRender.com.

Reduced L3MBTL1 levels inhibit cell division and cause G2/M phase arrest, indicating that the cells may be under replication stress. Although it is unclear how L3MBTL1 affects DNA replication, its inhibition affects the replication fork, implying that it also affects DNA replication. DDR is activated in the early stages of many cancers, with the pre-invasion stage being the most noticeable. Strong DDR activation is caused by the overexpression of activated oncogenes such as c-myc, H-Ras, Cdc25A, cyclin E1, and E2F1 in various cell types [28–30]. L3MBTL1 acts as a transcriptional repressor that promotes transcription following depletion. L3MBTL1 has previously been shown to interact with c-myc, and c-myc expression increases when L3MBTL1 is reduced [11]. This is supported by the increased expression of c-myc, H-Ras, Cdc25A, and E2F1 when L3MBTL1 is depleted, which was revealed in our RNA-seq analysis. According to another theory, H4K20Me2 is produced after L3MBTL1 is depleted before cells enter the G2/M phase. H4K20Me2 is found throughout the nucleus and works with 53BP1 to promote the DDR while inhibiting normal DNA replication [31]. Our findings back up these claims, indicating that L3MBTL1 contributes to the compacting of chromatin structure, reducing DNA damage repair, and increasing chromatin compactness in EGFR-TKI-acquired drug-resistant cells. After knocking down L3MBTL1, the chromatin structure relaxes, increasing the accessibility of DNA damage repair factors and thus increasing sensitivity to Osimertinib (Fig. 9). Osimertinib may also cause DNA damage. Osimertinib and L3MBTL1 knockdown increased apoptosis by inducing DNA damage.

ChIP-seq analysis revealed that L3MBTL1 is frequently associated with genes related to EGFR and its pathway genes as well as some EGFR-related bypass genes. It will be necessary to investigate the relationship between these genes and L3MBTL1, the effect on EGFR-TKI resistance, and the underlying molecular mechanism.

In conclusion, our research sheds light on the relationship between DDR and Osimertinib resistance. This may lead to the identification of new therapeutic targets for treating patients who have developed resistance to Osimertinib. We discovered that L3MBTL1 is a key factor in NSCLC with acquired resistance to Osimertinib. The depletion of L3MBTL1 significantly reduces drug resistance by regulating the DDR in vivo and in vitro. Our findings suggest that measuring L3MBTL1 expression could be a useful predictive biomarker for EGFR-TKI resistance.

## Materials and methods

### Cells and cell culture

The NSCLC cell line H1975 was obtained from the American Type Culture Collection. The H1975/AR cell line was kindly provided by Prof. Jin-Jian Lu from the University of Macau [32]. H1975/AR cells were exposed to 1 μM Osimertinib for an extended period to maintain resistance. Prof. Zhou at the Shanghai Pulmonary Hospital (Shanghai, China) donated PC-9 [33]. PC-9/OR cells were created by repeatedly exposing PC-9 cells to Osimertinib over time, followed by exposure to 0.5 μM Osimertinib to maintain resistance. All cells were grown in a humidified environment with 5% CO_2_ at 37 °C in RPMI 1640 medium containing 10% fetal bovine serum (Gibco, California, USA). All cells were STR-validated at Genechem (Shanghai, China) and tested for mycoplasma contamination.

### Reagents

Osimertinib (AZD9291), Etoposide, MG132, cycloheximide (CHX), and UNC669 were purchased from Selleck Chemicals LCC (Houston, USA). The following antibodies were used: γ-H2AX(Abcam, ab1175), KU70(Abcam, ab92450), KU80(Abcam, ab80592), H4K20Me1(Abcam, ab177188), H4K20Me2(Abcam, ab9052), L3MBTL1 (Abcam,ab92450, ab97304), GAPDH(proteintech 60004-1-lg), EGFR(Abcam, ab32562), 53BP1(CST, 4937S), p-EGFR(CST, 3777S), AKT(CST, 4691), P-AKT(proteintech, 66444-1-lg), MAPK(CST, 8690S), P-MAPK(CST, 9211S), BRCA1(CST, 9010T), RAD51(Abcam, ab133534), Ki-67(Abcam, ab16667), Histone H3(CST, 9715S), β-tubulin(proteintech, 66240-1-Ig), ubiquitin (CST,3933S), L3MBTL1 (Active Motif, USA), and RNF8 (proteintech, 14112-1-AP).

### siRNA interference and lentivirus infection

siRNA for human L3MBTL1 and 53BP1 were synthesized by Ribobio (Guangzhou, China) and transfected into cells with Lipofectamine 3000 (Life Technologies, New York, USA) following the manufacturer’s protocol. Lentiviral vectors encoding full-length L3MBTL1, short hairpin RNA of human L3MBTL1, and control sequences were purchased from Genechem (Shanghai, China). Western blot analysis and qPCR were used to confirm transfection efficiency.

### RNA extraction, reverse transcription, and quantitative real-time PCR

Total RNA was extracted with TRIzol (Thermo Fisher Scientific, MA, USA) and the RNeasy Mini Kit (Tiangen, Beijing, China), followed by reverse transcription to cDNA for quantitative real-time PCR experiments. The mRNA levels were normalized against GAPDH.

### Immunoblotting and immunoprecipitation

Immunoblotting analysis was performed following the procedure outlined in our previous study [34]. To perform immunoprecipitation, add the primary antibody to the cell lysate and rock overnight at 4 °C. Protein A beads were thoroughly washed with lysis buffer, and the supernatant was analyzed using western blotting. The relative protein levels were normalized to GAPDH, β-tubulin, and Histone H3.

### ROS generation assay

ROS levels were determined using the Reactive Oxygen Species Assay Kit (Beyotime, Shanghai, China) according to the manufacturer’s specifications. A mock control was given to the cells during the logarithmic growth phase. Cells were treated with vehicle or Osimertinib for 24 h at 37 °C before being incubated with 10 μM DCFH-DA in a serum-free medium at 37 °C for 20 min. The cells were then detected using flow cytometry and analyzed with FlowJo software.

### Colony formation assay, CCK-8 assay, and Transwell invasion assay

The colony formation assay and CCK-8 assay [34]. And the Transwell invasion assay [35] were performed as described previously.

### Comet assay

The Comet assay was done according to the manufacturer’s instructions (Abcam, UK). Briefly, cells were seeded into 6-well plates and treated with Osimertinib for 24 h. The cells were then stained with Vista Green DNA. After imaging with a fluorescence microscope, the tail distance and Comet score were determined.

### NHEJ/HR assay and micrococcal nuclease digestion assay

To perform the NHEJ assay, cells were transfected with pDRGFP (Addgene #26475) or pimEJ5GFP (Addgene #44026) [36] which were then linearized with *EcoRV* and *XhoI* (TAKARA, Japan). The linearized plasmids were transfected into cells with Lipofectamine 3000 according to the manufacturer’s instructions. The cells were incubated for 24 h and examined by fluorescence microscopy. The micrococcal nuclease digestion assay was performed using the micrococcal nuclease digestion kit (NEB, MA, USA) according to the manufacturer’s protocol. Briefly, the cells were centrifuged and resuspended in 100 μL of either MNase-containing or MNase-free MNase Buffer at room temperature for 4 min. The cell pellets were collected by centrifugation and DNA was extracted with the TIANamp Genomic DNA Kit (TIANGEN, Beijing, China) before being subjected to horizontal electrophoresis.

### Immunofluorescence staining and immunohistochemistry

The immunofluorescence analysis was performed as explained in a prior study [35]. γ-H2AX (diluted 1 : 200; Abcam, UK) and 53BP1 (diluted 1:200; Abcam, Cambridge, UK) were used as primary antibodies and immunohistochemistry was performed as previously described [34].

### RNA-Seq analysis

RNA-seq was performed by the Beijing Genomics Institute (BGI, Shenzhen, China) and analyzed using the web-based platform Dr. Tom 3.0 (BGI, Shenzhen, China). The raw sequence data reported in this paper have been deposited in the Genome Sequence Archive (Genomics, Proteomics & Bioinformatics 2021) in National Genomics Data Center (Nucleic Acids Res 2022), China National Center for Bioinformation/Beijing Institute of Genomics, Chinese Academy of Sciences (GSA-Human: HRA005731), which is publicly accessible at https://ngdc.cncb.ac.cn/gsa-human [37, 38].

### Chromatin immunoprecipitation and sequencing analysis

Chromatin immunoprecipitation was performed with the Chromatin Immunoprecipitation (ChIP) Assay Kit (Millipore, MA, USA) according to the manufacturer’s protocol. The chromatin/antibody complex was pulled down using the protein G agarose beads included in the kit. After the various washing steps, the chromatin was eluted. Quality control and library construction for chromatin immunoprecipitation sequencing (ChIP-Seq) using Illumina Hiseq were performed by Wuhan Seqhealth Technology Co., Ltd (Wuhan, China). The raw sequence data reported in this paper have been deposited in the Genome Sequence Archive (Genomics, Proteomics & Bioinformatics 2021) in the National Genomics Data Center (Nucleic Acids Res 2022), China National Center for Bioinformation / Beijing Institute of Genomics, Chinese Academy of Sciences (GSA-Human: HRA005732), which is publicly publicly accessible at https://ngdc.cncb.ac.cn/gsa-human [37, 38].

### In vivo subcutaneous xenografts

The nude mice and feeding conditions were the same as previously described [35]. The Committee of Animal Care and Use of Tianjin Medical University General Hospital authorized all experiments with animals in this study. H1975 or H1975/AR cells (2 × 10^6^) were injected subcutaneously into nude mice’s left groins. When the tumor volume reached about 200 mm^3^, the mice were randomly divided into 2 groups of 5, and each group was given Osimertinib (2.5 mg/kg/day) for 2 weeks. For the L3MBTL1 knocking down experiment, H1975/AR sh-NC and H1975/AR sh-L3MBTL1 cells (2 × 10^6^) were injected subcutaneously into nude mice’s left groin. Once the tumor volume reached 150–200 mm^3^, the mice were randomly divided into 5 groups of five mice each and fed double-distilled water, Osimertinib (2.5 mg/kg/day), or Etoposide (5 mg/kg/first 5 days) for 2 weeks. The animals were cared for according to the guidelines of the Tianjin Medical University Animal Care and Use Committee.

### Functional annotation

GSEA was used to investigate the relationship between gene expression in a gene set and the class labels [39]. The R package clusterProfiler was used to investigate enrichment pathways. The online Database DAVID (http://david.ncifcrf.gov/) [40] and BiNGO [41] (plugin for Cytoscape [42]) were used for annotation, visualization, and integrated discovery in addition to GO and KEGG pathway analysis. A Heatmap was created using the R package heatmap.

### Statistical analysis

This study’s experimental assays were repeated at least three times, with data presented as mean ± standard deviation (SD). All data was analyzed with GraphPad Prism 8.0.1. Chi-square and Student’s t-tests were used to assess the significance of the difference between the two groups. Nude mice tumor volume and CCK-8 assay results were assessed for differences using a two-way ANOVA. P < 0.05 was considered statistically significant.

## Supplementary information

Supplement Figure1,2

Western blot origin

## Data Availability

All data needed to evaluate the conclusions in this study are present in this article and Supplementary Materials. Additional data related to this paper may be requested from the authors.
