# Supplementary material for: L3MBTL1, a polycomb protein, promotes Osimertinib acquired resistance through epigenetic regulation of DNA damage response in lung adenocarcinoma
Source: Cell Death Dis. 2024 Sep 4;15(9):649. doi: 10.1038/s41419-024-06796-2 (PMC11374981; doi:10.1038/s41419-024-06796-2)
Supplement: Supplementary file 1 — Supplement Figure1,2 [file 41419_2024_6796_MOESM1_ESM.docx]

| 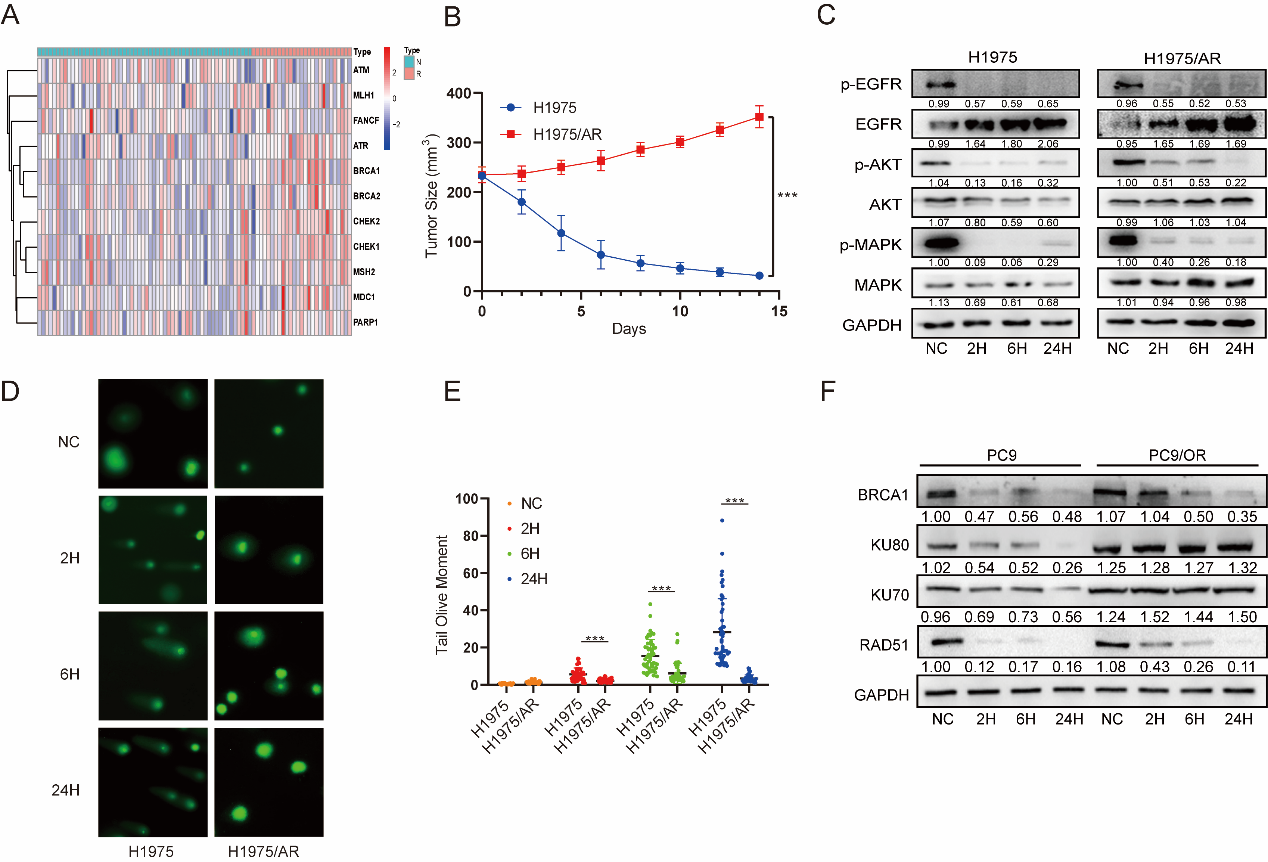 |
| --- |

**Figure S1.** A heatmap analysis of lung adenocarcinoma patient tissues after recurrence shows increased expression of genes associated with DNA damage response. (B) Nude mice were subcutaneously implanted with H1975 and H1975/AR cell lines, respectively, and then treated with Osimertinib for 2 weeks after the tumor size reached 200mm^3^. The tumor volume was measured and recorded. Results are presented as mean ± standard deviation. (C)Immunoblotting was used to determine the effect of Osimertinib treatment in H1975 and H1975/AR on the expression of EGFR and downstream pathway proteins over time. (D, E) H1975 and H1975/AR were treated with Osimertinib over time, DNA damage was measured by Comet assay, tail olive moment analysis was detected by Cometscore (mean ± SD, n = 3, *P < 0.05, **P < 0.01, ***P < 0.001). (mean ± SD, n = 3, *P < 0.05, **P < 0.01, ***P < 0.001). (F) Immunoblotting was used to determine the effect of Osimertinib treatment in PC9 and PC9/OR on the expression of NHEJ and HR key proteins over time.

| 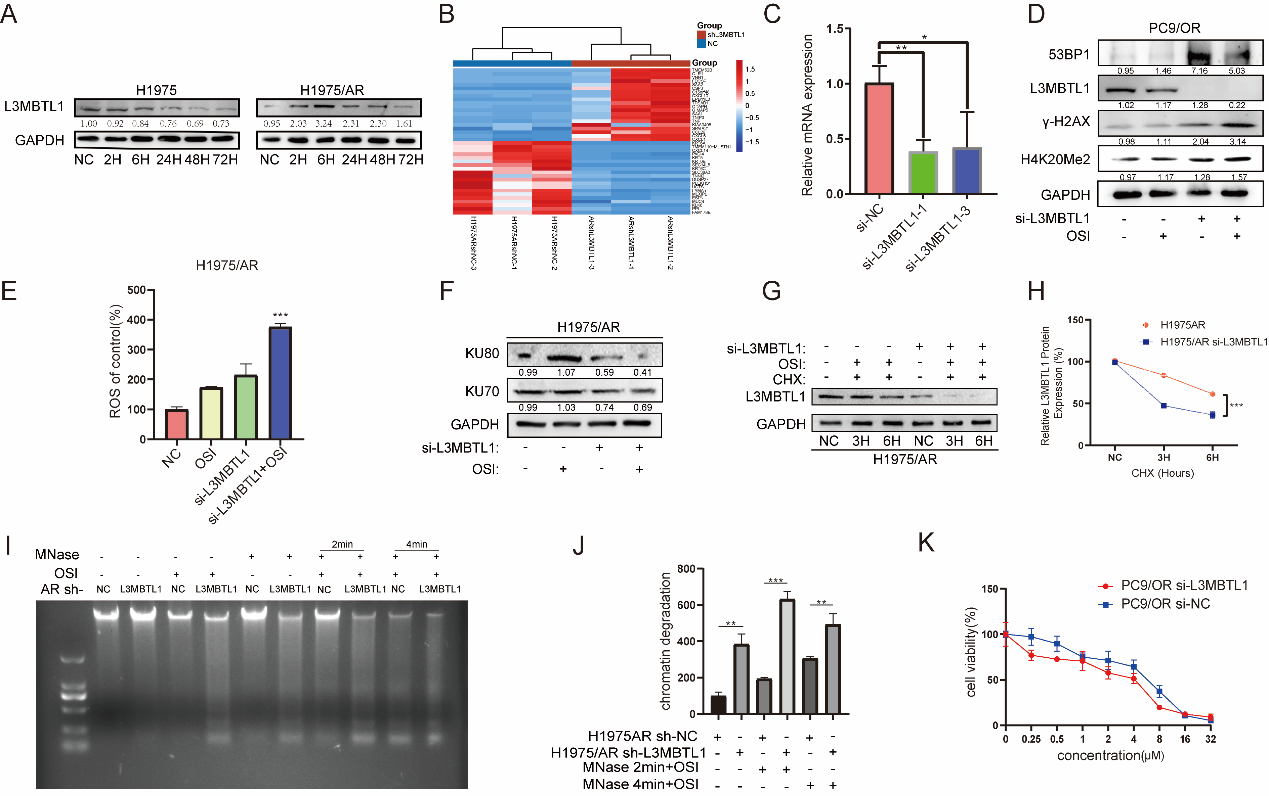 |
| --- |

**Figure S2** (A) Immunoblotting was used to assess the effect of Osimertinib treatment in H1975 and H1975/AR on the expression of L3MBTL1 over time. (B) Heatmap of the genes that differed in expression between H1975/AR sh-L3MBTL1 cells and H1975/AR sh-NC cells. (C) Using qRT-PCR to measure the mRNA levels of transfection efficiency of Si-L3MBTL1-1 and Si-L3MBTL1-3 in H1975/AR (mean ± SD, n = 3, *P < 0.05, **P < 0.01, ***P < 0.001). (D) PC9 and PC9/OR were transfected into si-L3MBTL1-3, with or without treated with Osimertinib. 53BP1, L3MBTL1, γ-H2AX, and H4K20Me2 protein levels were evaluated by immunoblotting. (E) After transfection with si-L3MBTL1-3 or scrambled siRNA for 48h, the H1975AR cells were treated with DMSO or Osimertinib, and the level of ROS was examined by DCFH-DA staining. (mean ± SD, n = 3, *P < 0.05, **P < 0.01, ***P < 0.001). (F) After transfected with si-L3MBTL1-3 or scrambled siRNA for 48h, the H1975AR cells were treated with DMSO or Osimertinib, KU70, and KU80 protein expression were identified by immunoblotting, and relative density was measured using Image J (mean ± SD, n = 3, *P < 0.05, **P < 0.01, ***P < 0.001). (G, H) After transfected with si-L3MBTL1-3 or scrambled siRNA for 48h, the H1975AR cells were treated with Osimertinib and CHX alone or combined over time, L3MBTL1 protein expression was identified by immunoblotting, and relative density was determined using Image J (mean ± SD, n = 3, *P < 0.05, **P < 0.01, ***P < 0.001). (I, J) Micrococcal Nuclease digestion assay determined the extent of MNase sensitivity in H1975/AR sh-NC AND H1975/AR sh-L3MBTL1 cells with Osimertinib treated. Relative density was evaluated using Image J (mean ± SD, n = 3, *P < 0.05, **P < 0.01, ***P < 0.001). (K) CCK-8 assays in PC9/OR cells after transfection with si-NC or si-L3MBTL1-3 for the indicated number of hours, followed by treatment with DMSO or Osimertinib. (H, I) After cells transfected with si-NC, si-L3MBTL1-1, or si-L3MBTL1-3 48h as indicated, with treated for DMSO or Osimertinib, the invasion was measured using a transwell assay in H1975/AR. (mean ± SD, n = 3, *P < 0.05, **P < 0.01, ***P < 0.001).
