# Supplementary material for: L3MBTL1, a polycomb protein, promotes Osimertinib acquired resistance through epigenetic regulation of DNA damage response in lung adenocarcinoma
Source: Cell Death Dis. 2024 Sep 4;15(9):649. doi: 10.1038/s41419-024-06796-2 (PMC11374981; doi:10.1038/s41419-024-06796-2)
Supplement: Supplementary file 2 — Western blot origin [file 41419_2024_6796_MOESM2_ESM.docx]

| Figure | Origin photo |
| --- | --- |
| 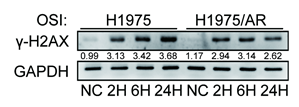  Figure 1D H1975&AR OSI γ-H2AX | 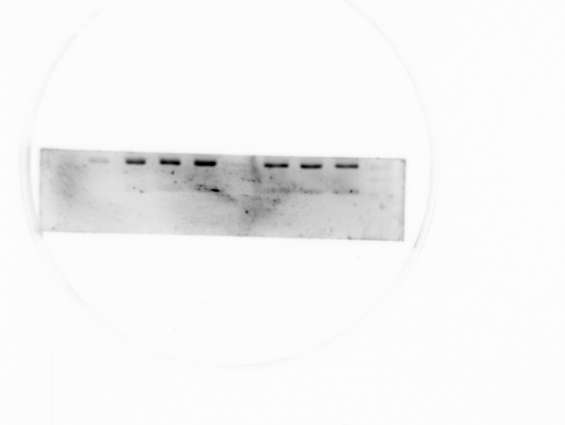 |
| 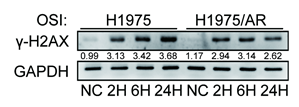  Figure 1D H1975&AR OSI GAPDH | 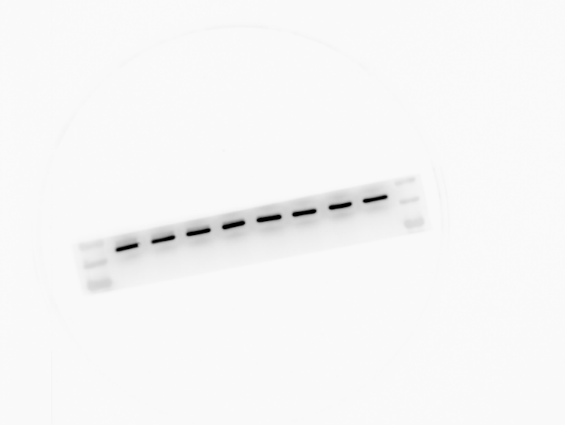 |
| 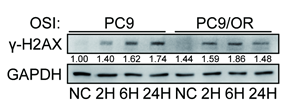  Figure 1D PC9&OR OSI γ-H2AX | 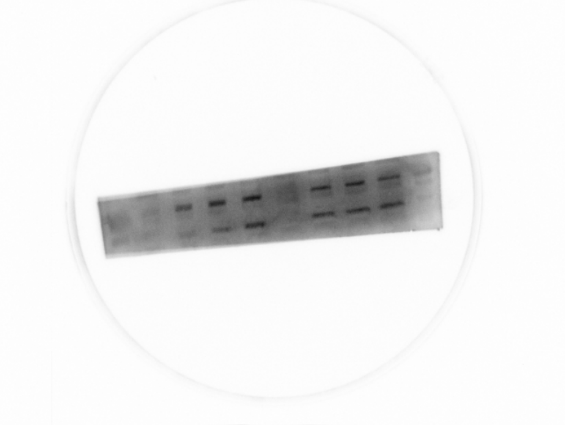 |
| 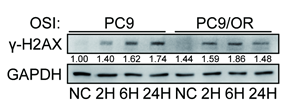  Figure 1D PC9&OR OSI GAPDH | 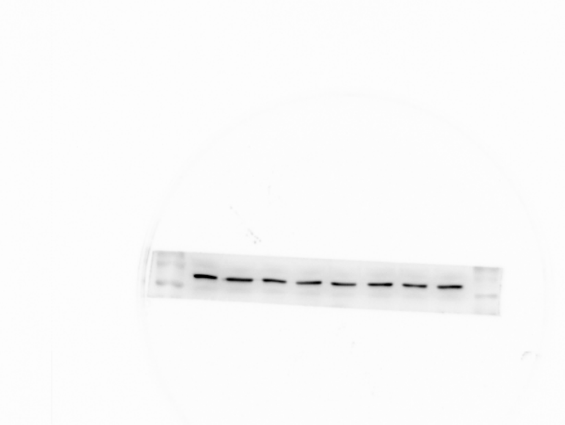 |

| 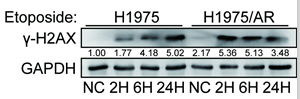  Figure 1E H1975&AR Etop γ-H2AX | 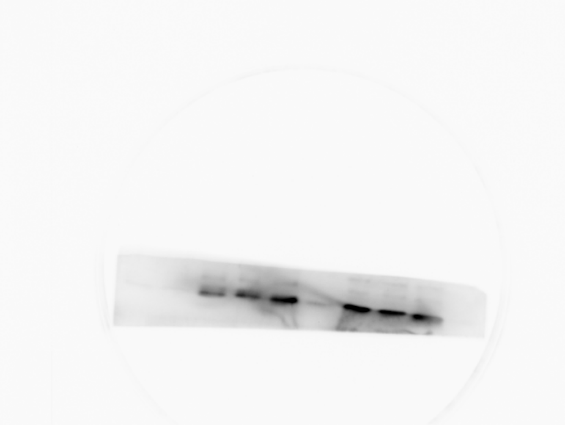 |
| --- | --- |
| 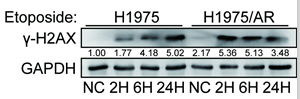  Figure 1E H1975&AR Etop GAPDH | 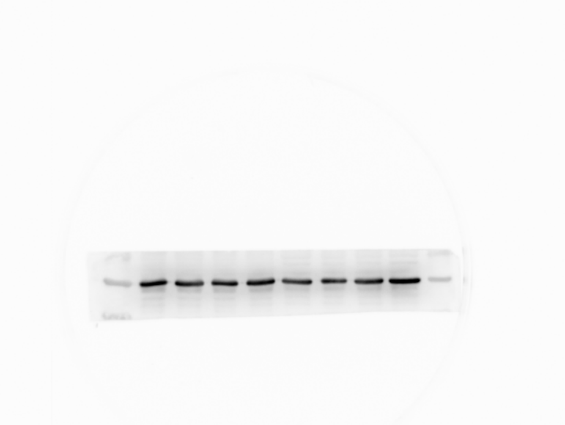 |
| 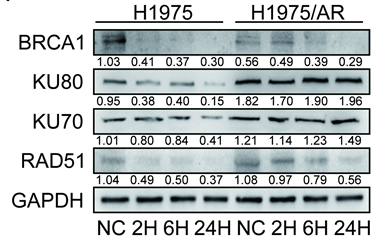  Figure 2A H1975&AR BRCA1 | 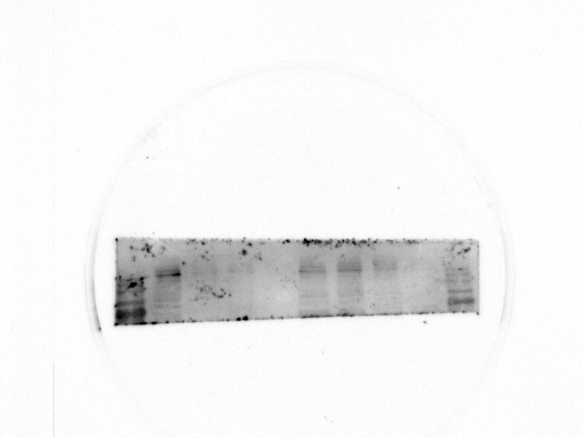 |
| 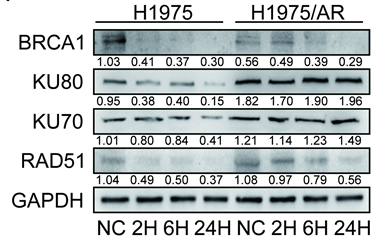  Figure 2A H1975&AR KU80 | 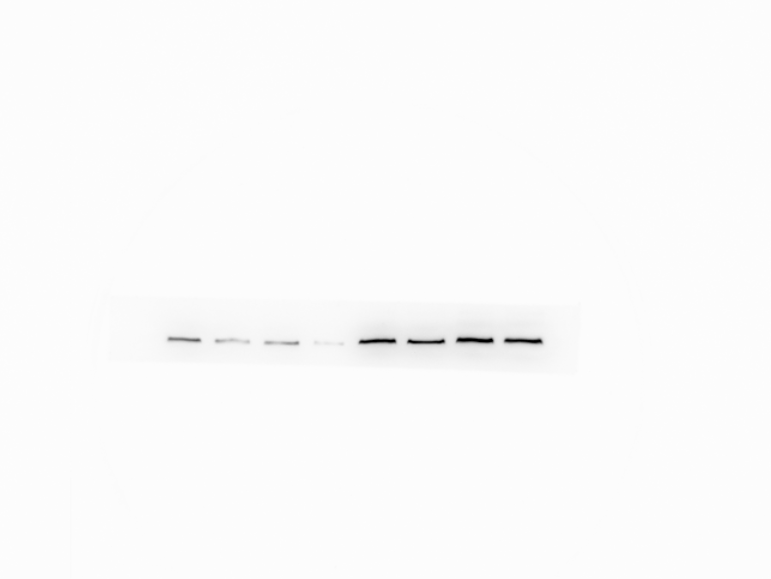 |
| 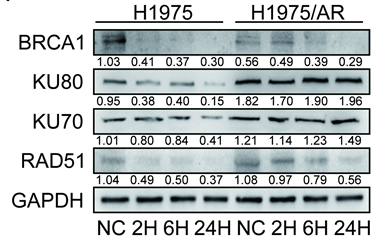  Figure 2A H1975&AR KU70 | 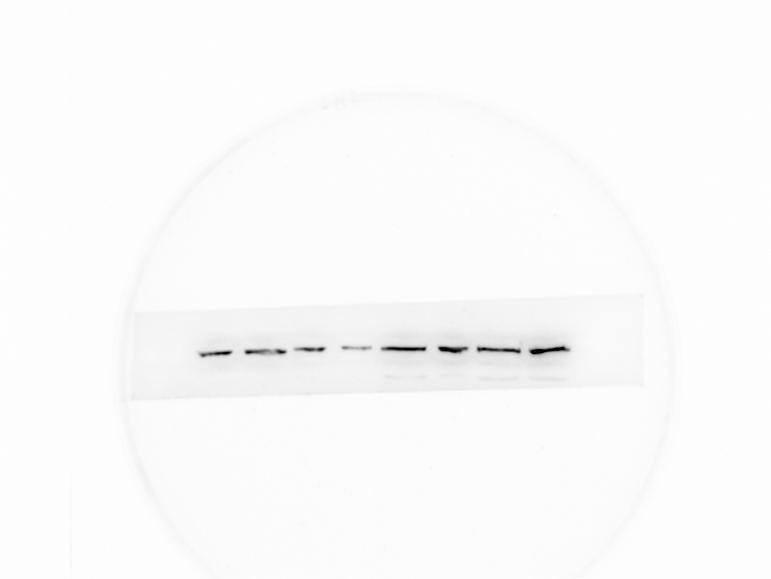 |
| 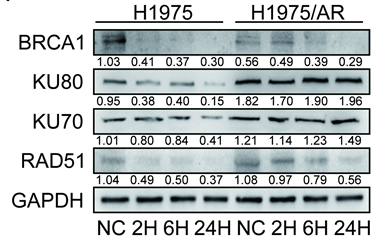  Figure 2A H1975&AR RAD51 | 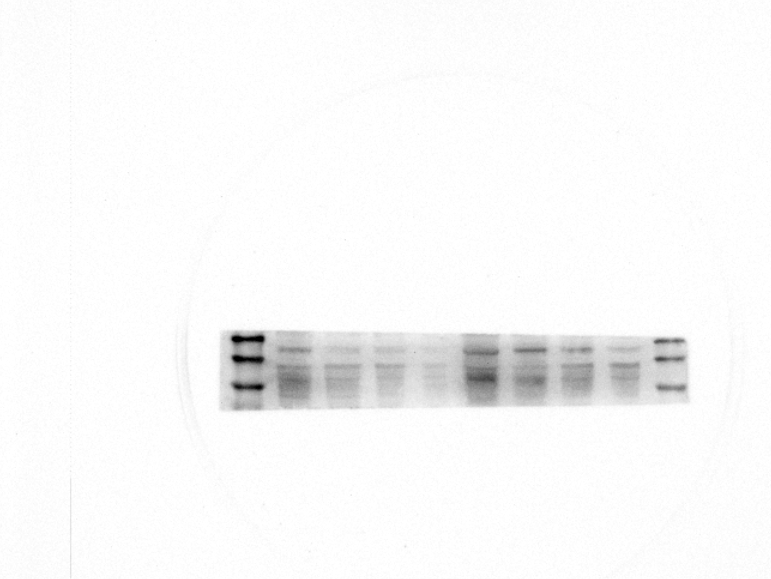 |
| 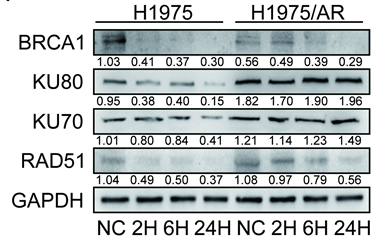  Figure 2A H1975&AR GAPDH | 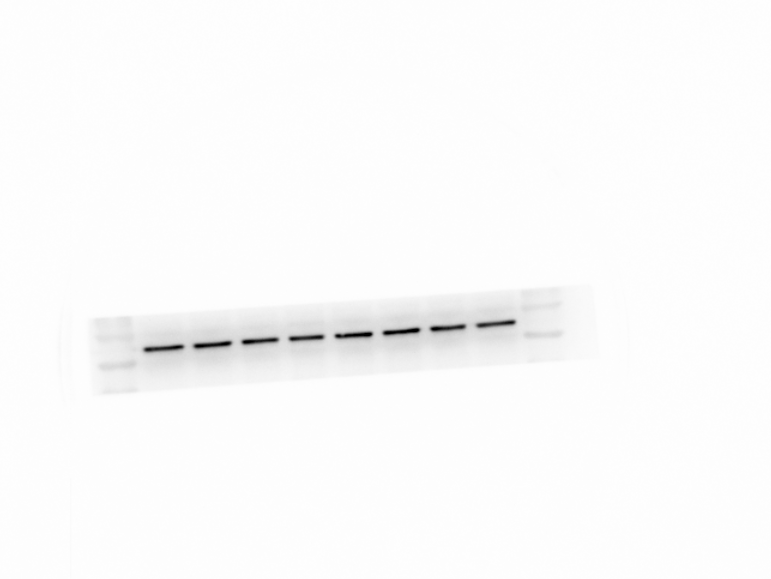 |
| 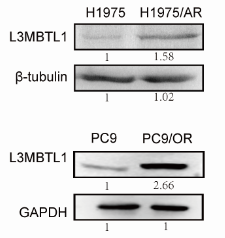  Figure 3A H1975&AR L3MBTL1 | 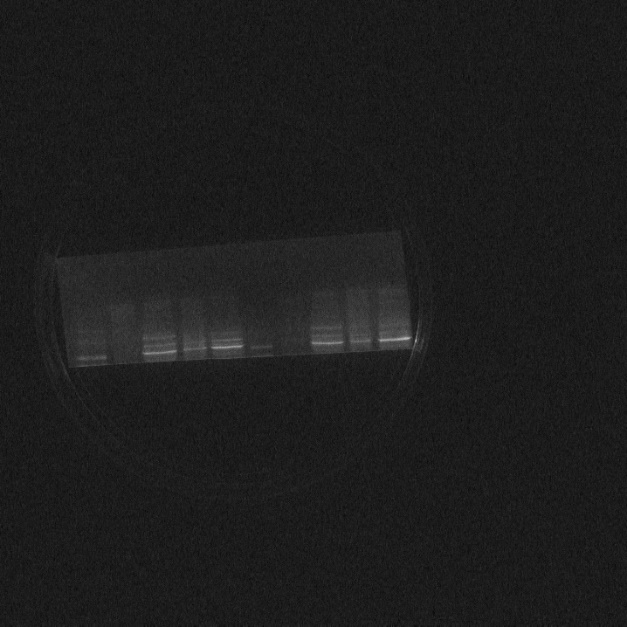4 groups repeated  **1**  **2**  **3**  **4** |
| 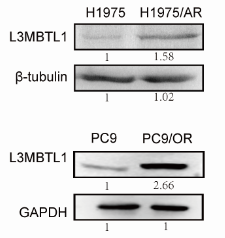  Figure 3A H1975&AR tubulin | 4 groups repeated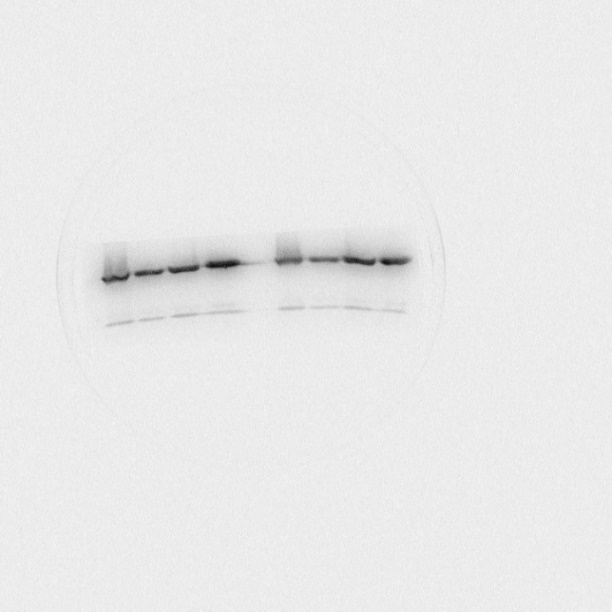  **4**  **3**  **2**  **1** |
| 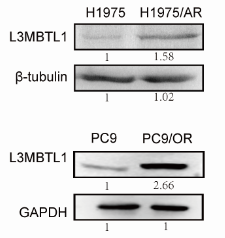.  Figure 3A PC9&OR L3MBTL1 | 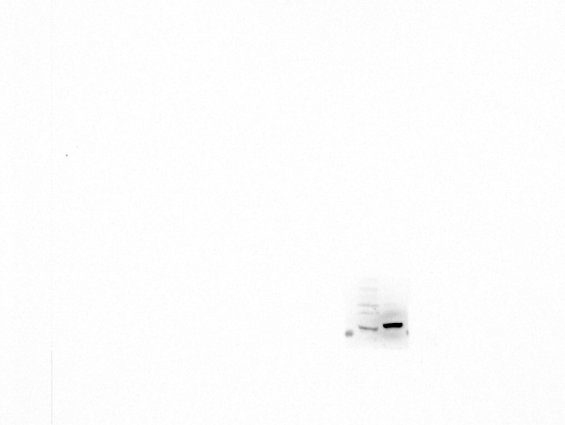 |
| 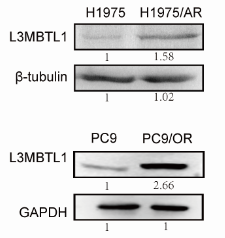Figure 3A PC9&OR GAPDH | 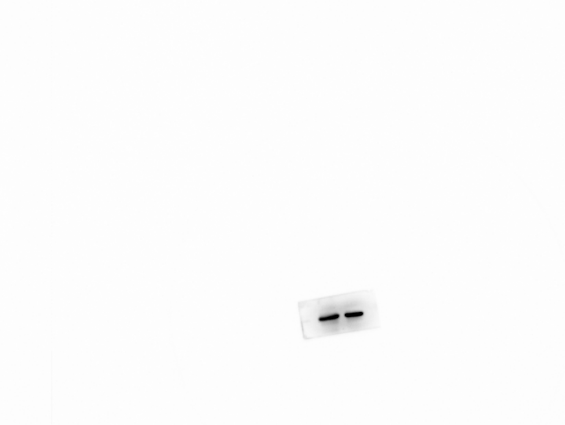 |
| 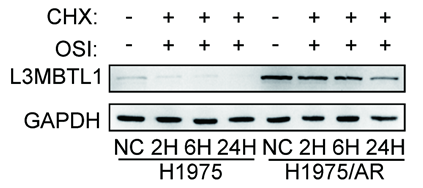Figure 3B H1975&AR L3MBTL1 | 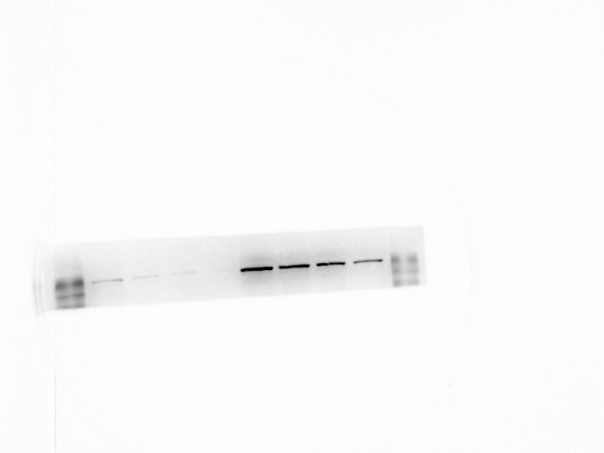 |
| 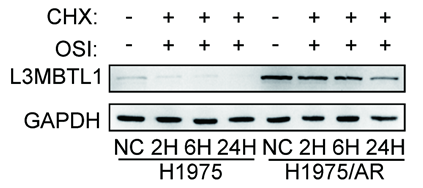Figure 3B H1975&AR GAPDH | 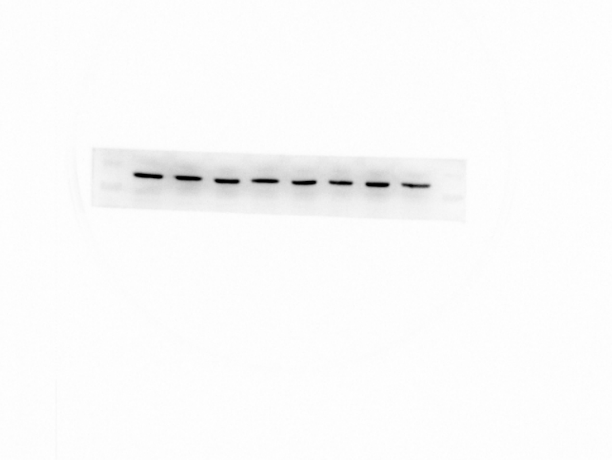 |
| 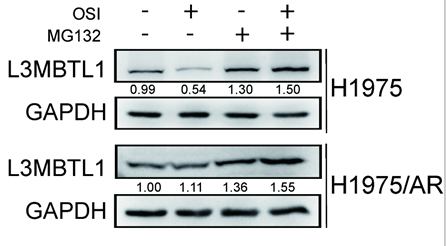Figure 3D H1975 L3MBTL1 |  |
| 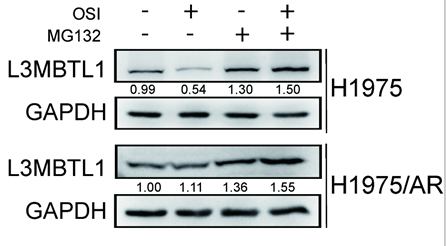Figure 3D H1975 GAPDH | 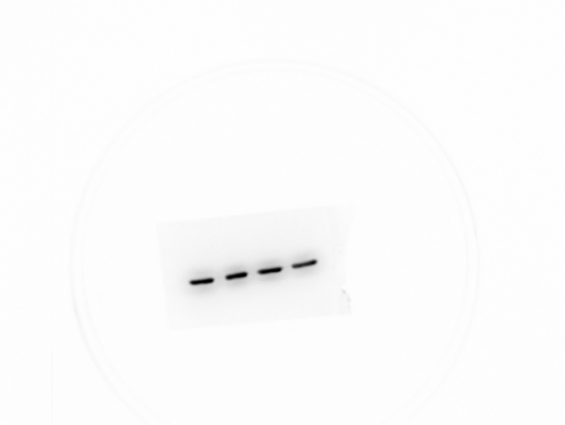 |
| 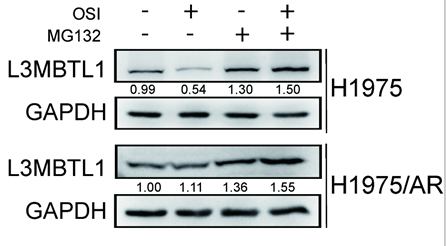Figure 3D H1975/AR L3MBTL1 | 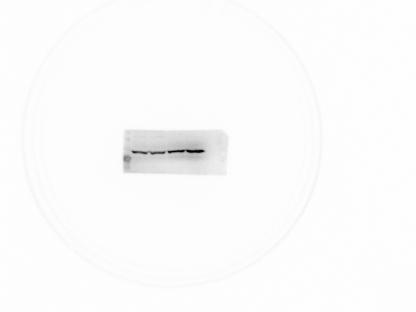 |
| 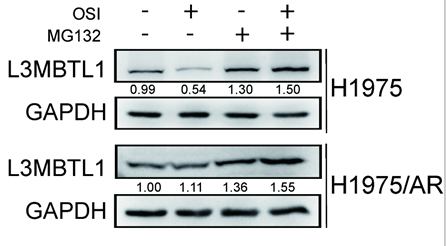  Figure 3D H1975/AR GAPDH | 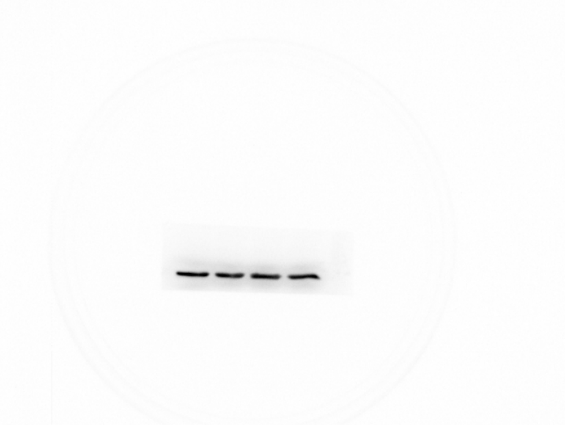 |
| 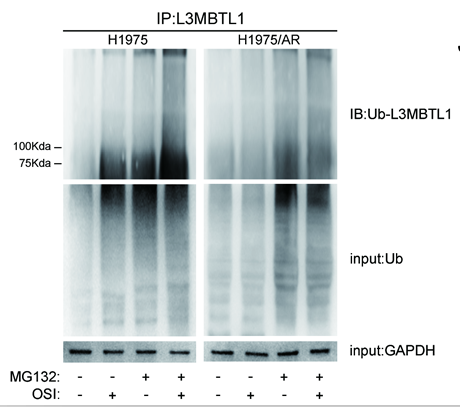  Figure 3E IP IB-Ub H1975 | 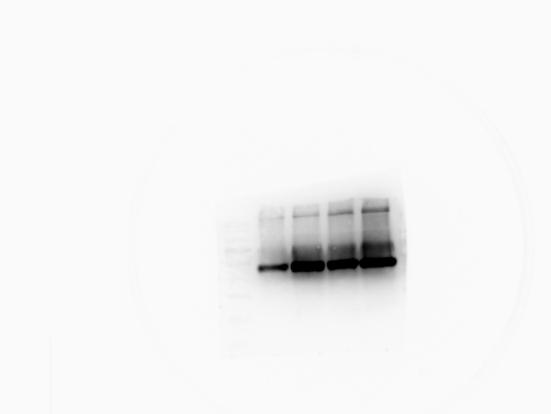 |
| 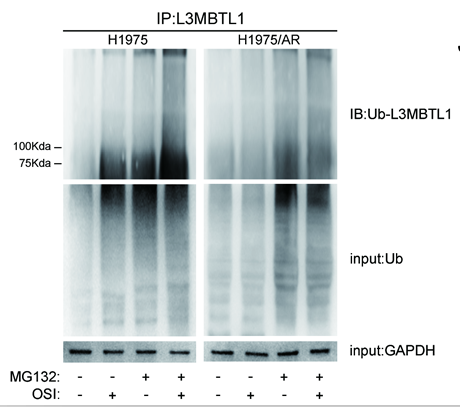  Figure 3E IP IB-Ub H1975/AR | 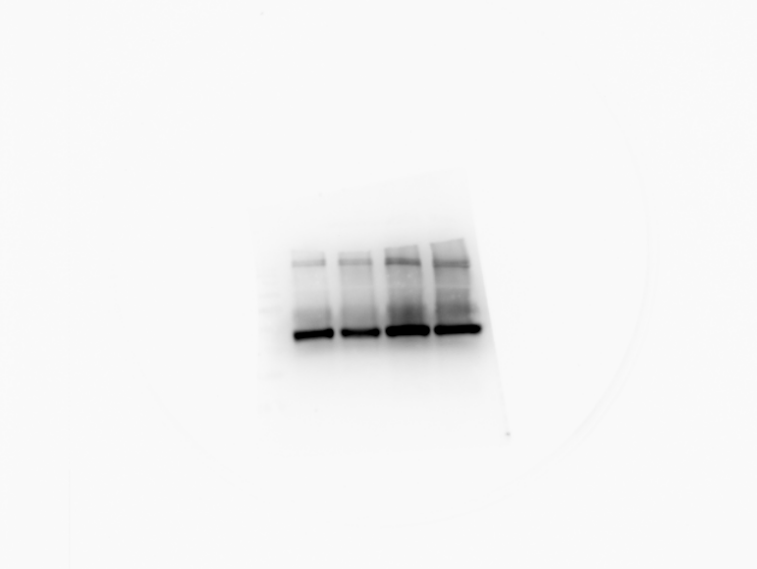 |
| 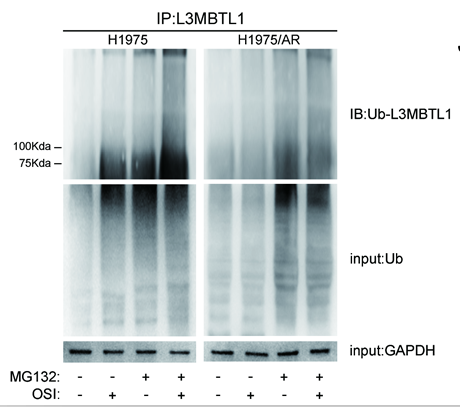  Figure 3E Input H1975 Ub | 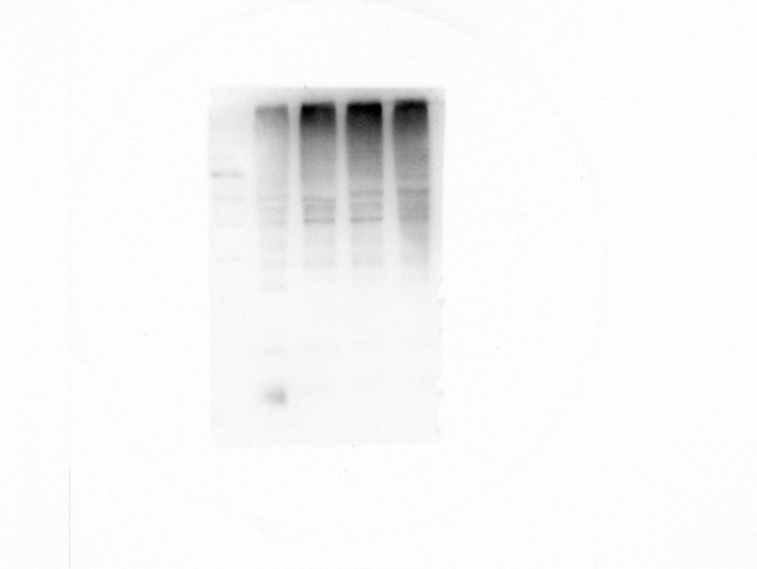 |
| 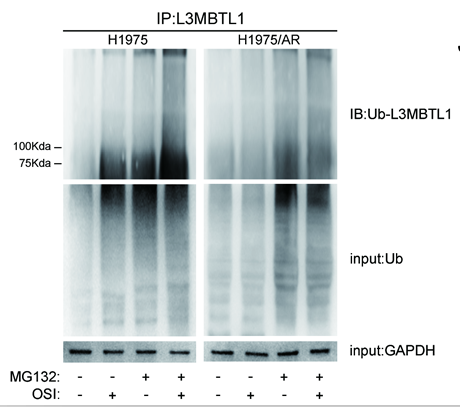  Figure 3E Input H1975/AR Ub | 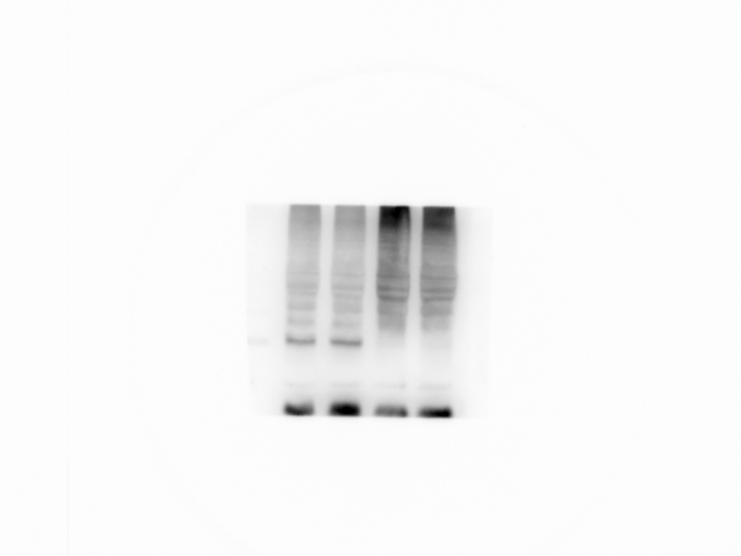 |
| 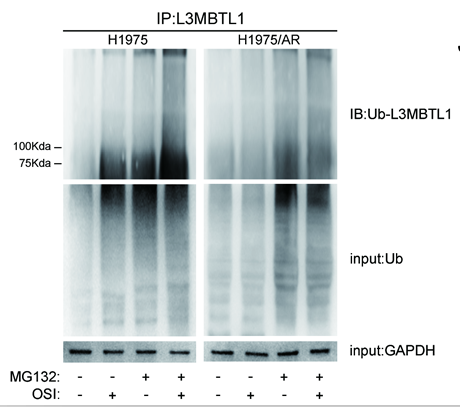  Figure 3E GAPDH H1975 | 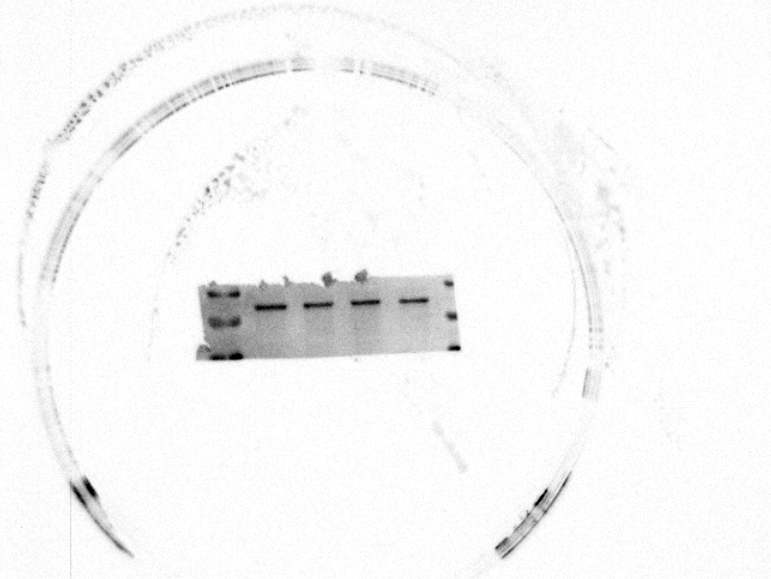 |
| 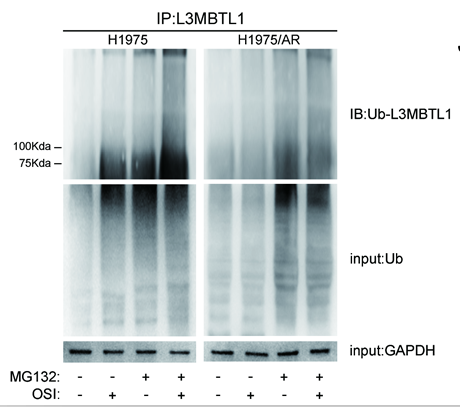  Figure 3E GAPDH H1975/AR | 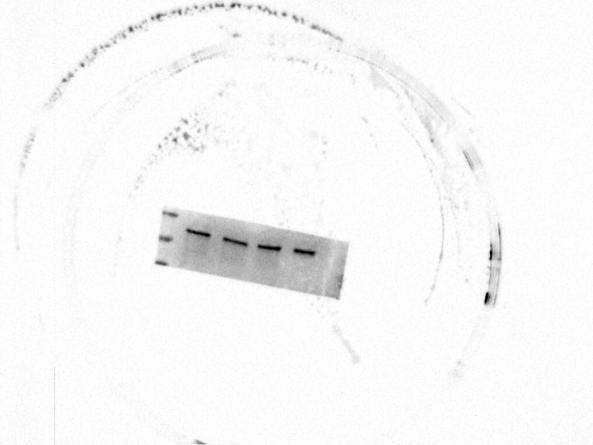 |
| 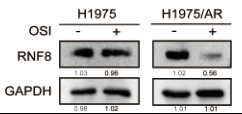  Figure 3G H1975 RNF8 | 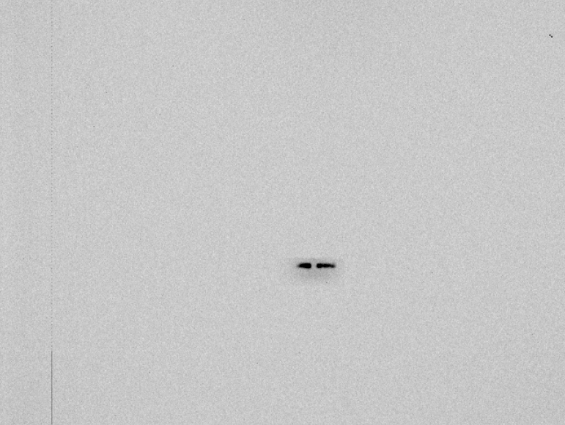 |
| 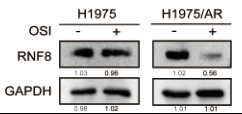  Figure 3G H1975/AR RNF8 | 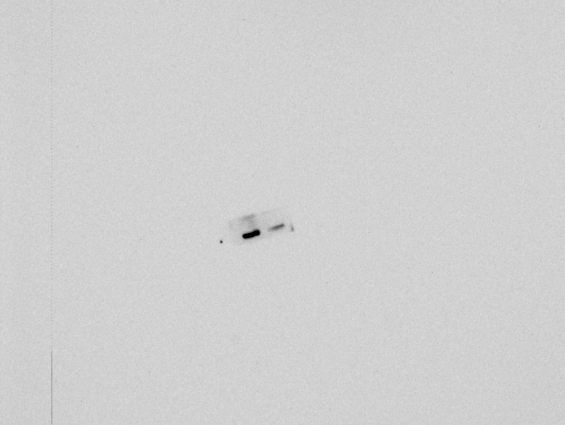 |
| 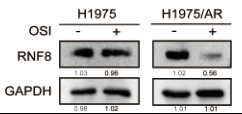  Figure 3G H1975 GAPDH | 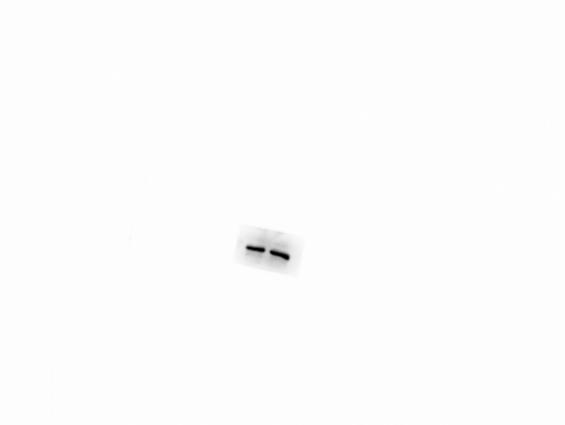 |
| 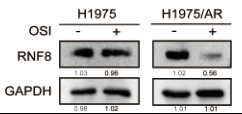  Figure 3G H1975/AR GAPDH | 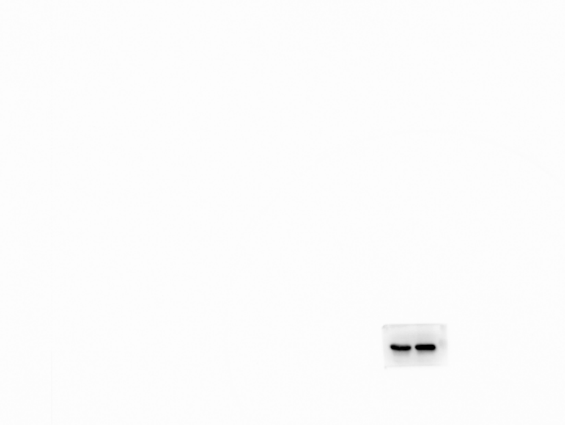 |
| 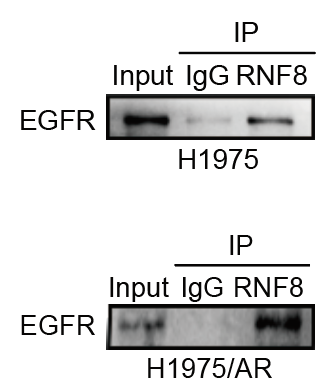  Figure 3H H1975 | 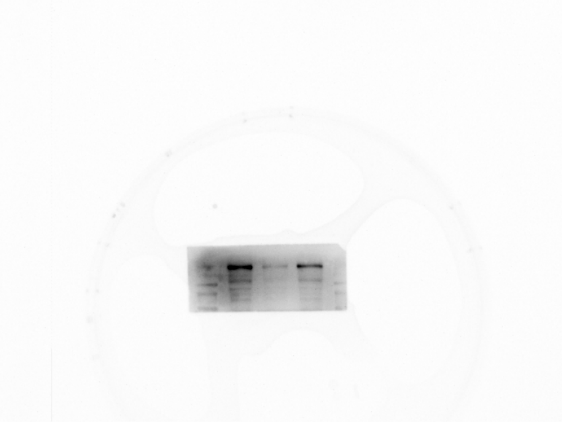 |
| 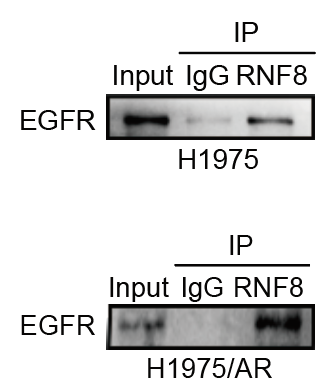  Figure 3H H1975/AR | 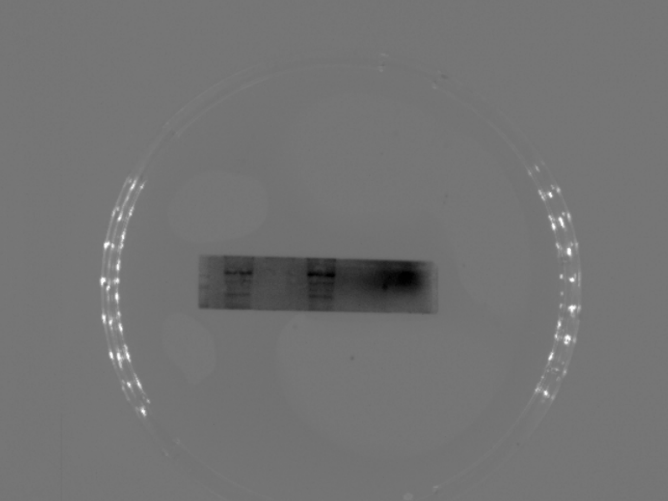 |
| 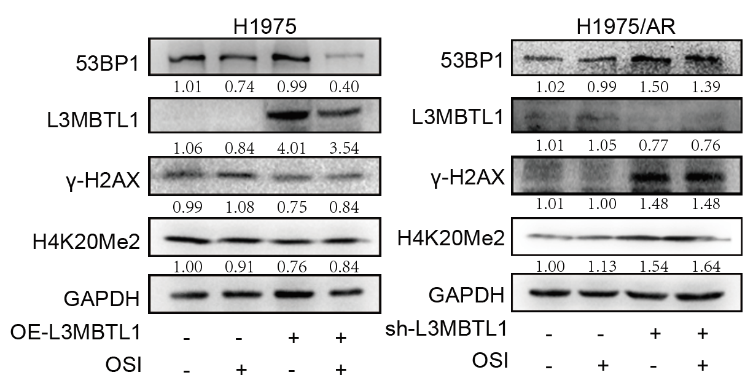­­  Figure 4B H1975 53BP1 | 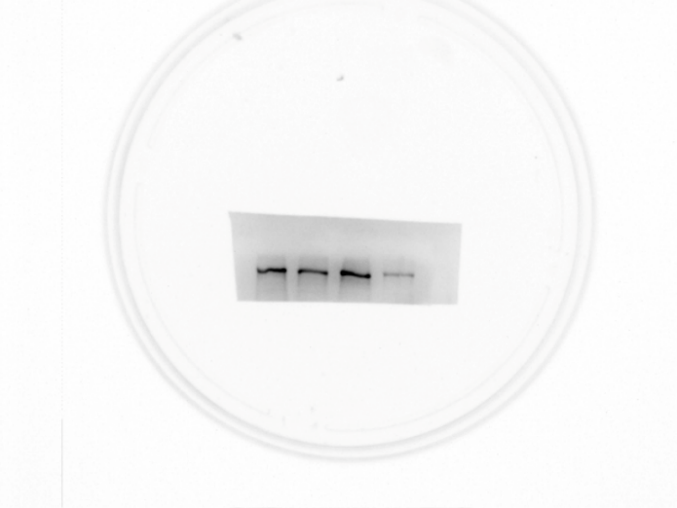 |
| 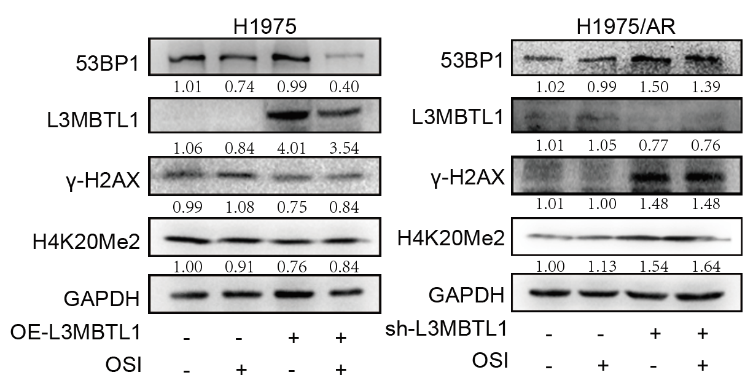­­  Figure 4B H1975 L3MBTL1 | 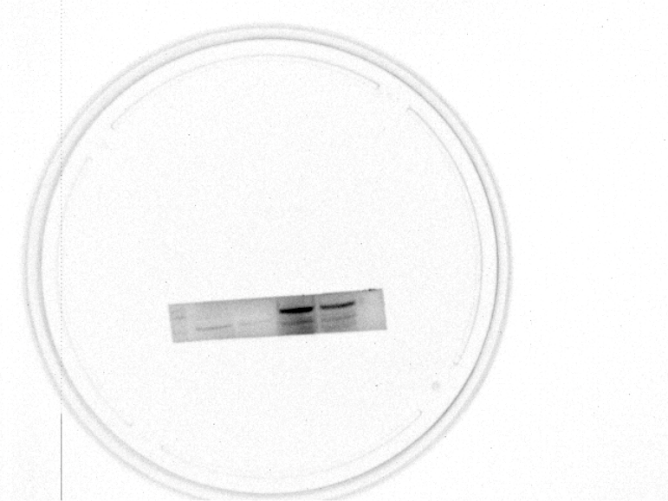 |
| 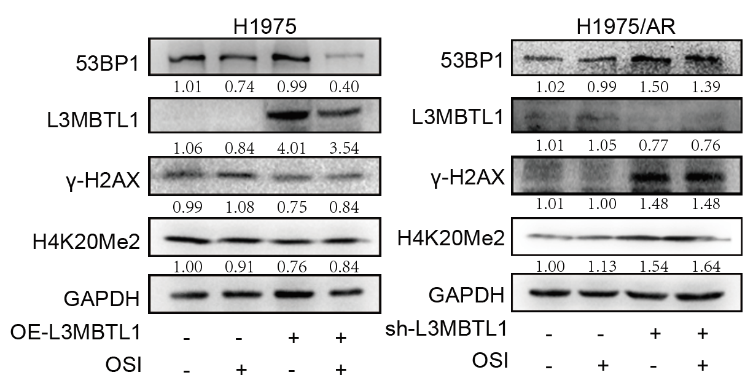­­  Figure 4B H1975 γ-H2AX | 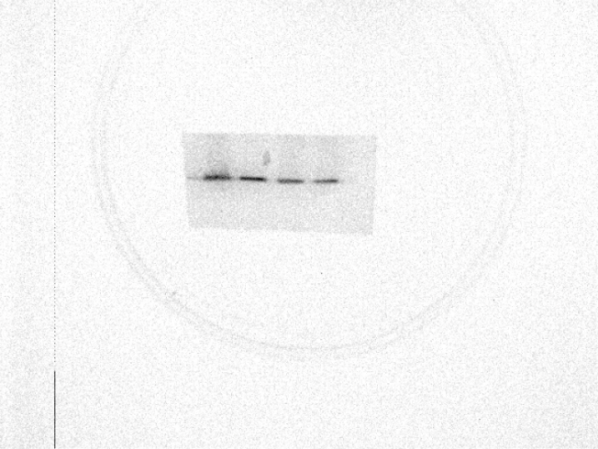 |
| 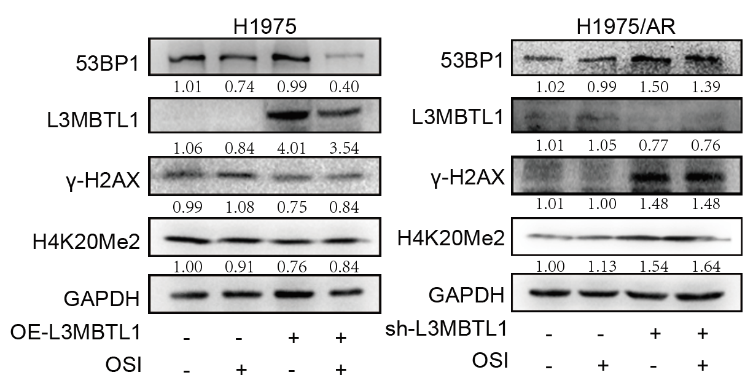­­  Figure 4B H1975 H4K20ME2 | 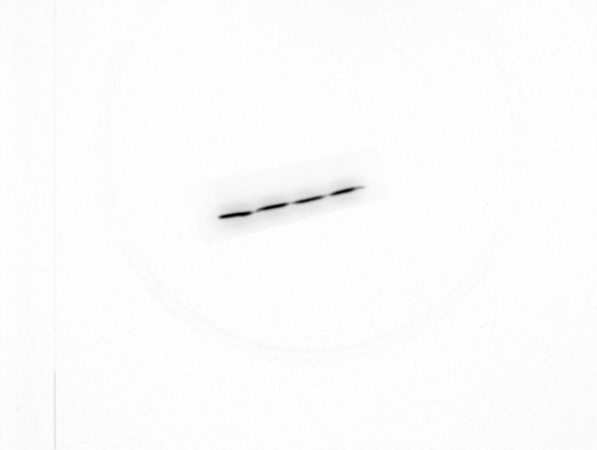 |
| 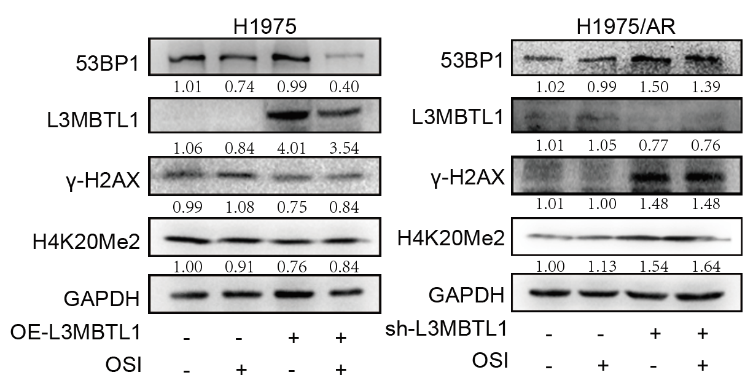­­  Figure 4B H1975 GAPDH | 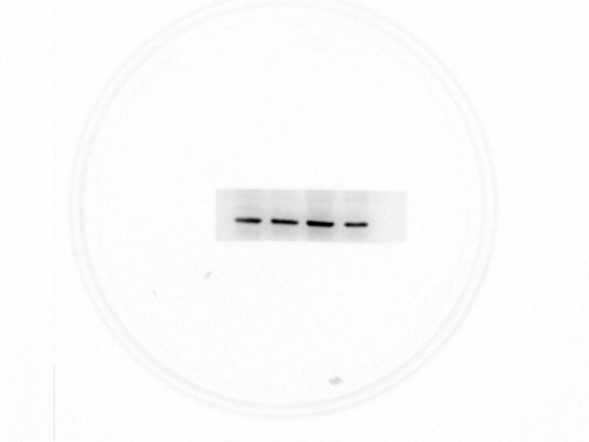 |
| 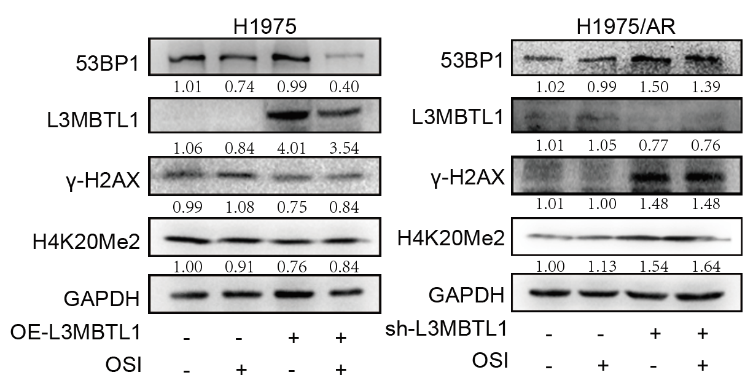­­  Figure 4B H1975/AR 53BP1 | 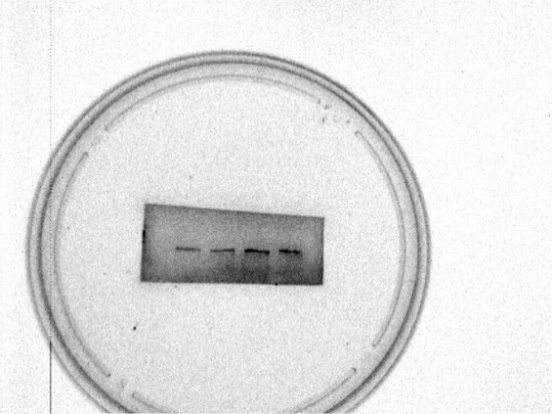 |
| 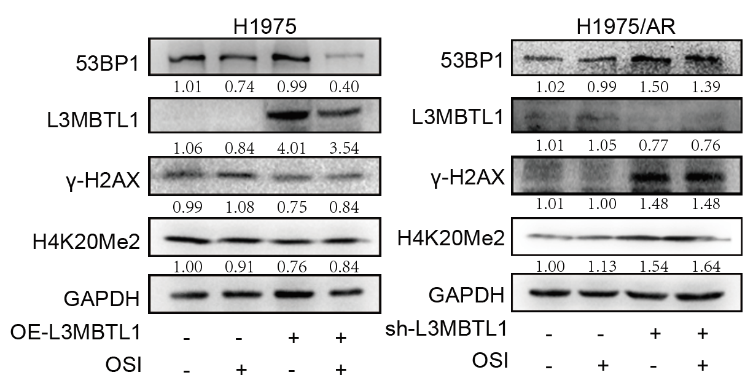­­  Figure 4B H1975/AR L3MBTL1 | 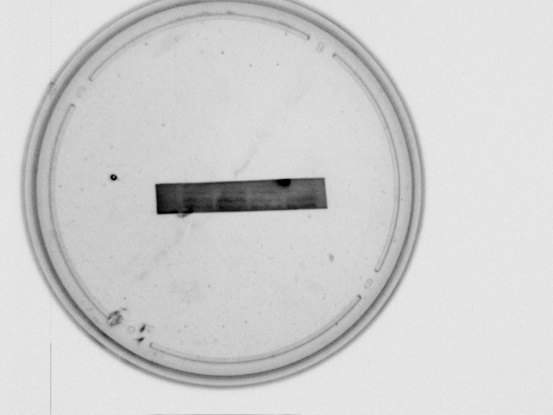 |
| 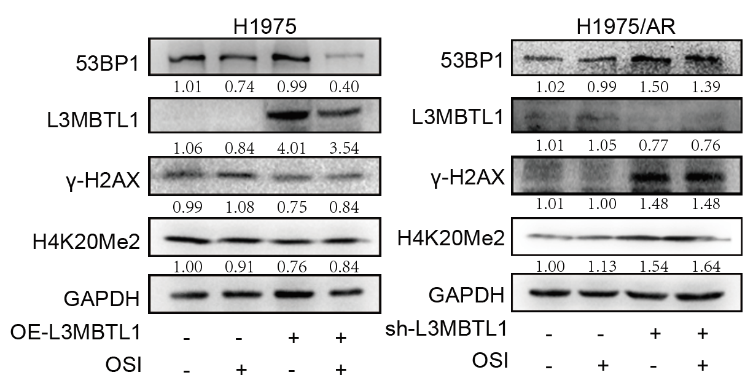­­  Figure 4B H1975/AR γ-H2AX | 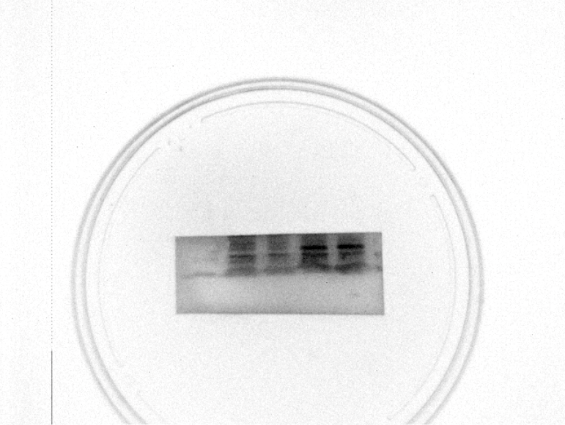 |
| 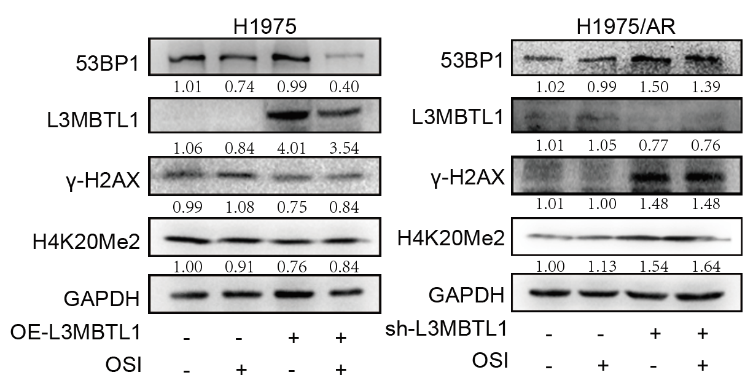­­  Figure 4B H1975/AR H4K20ME2 | 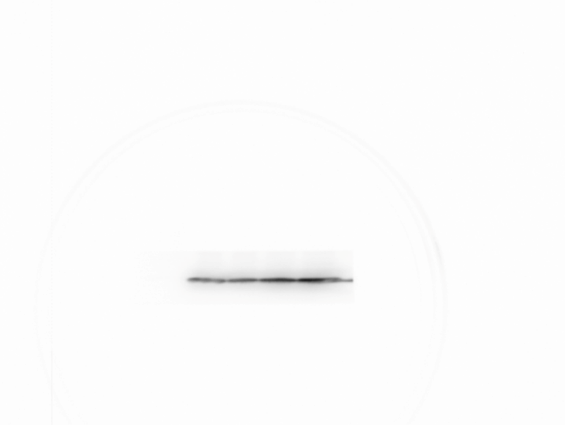 |
| 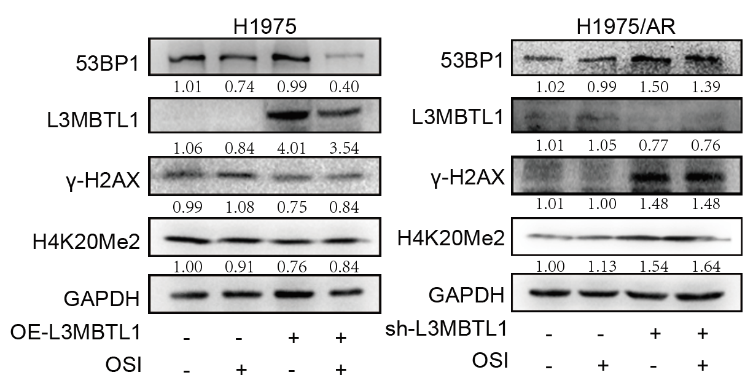­­  Figure 4B H1975/AR GAPDH | 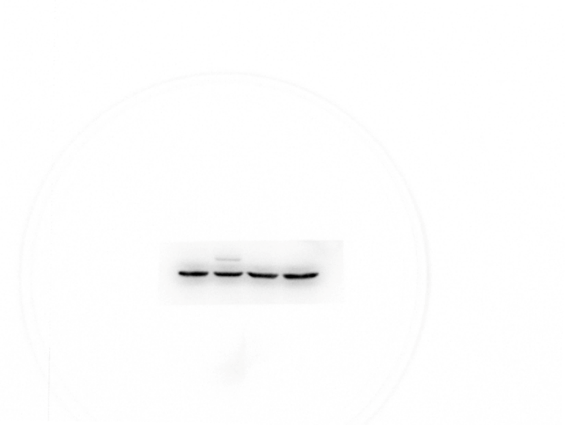 |
| 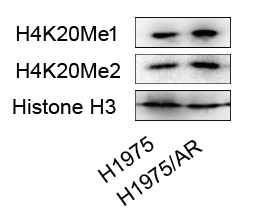  Figure 7A H4K20Me1 | 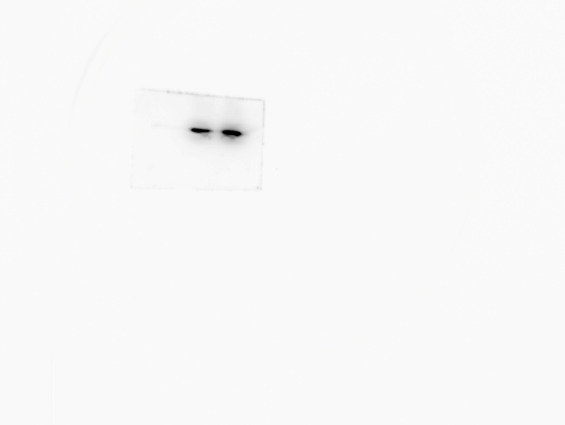 |
| 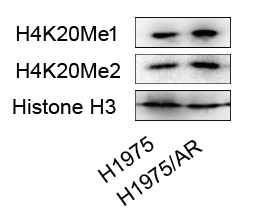  Figure 7A H4K20Me2 | 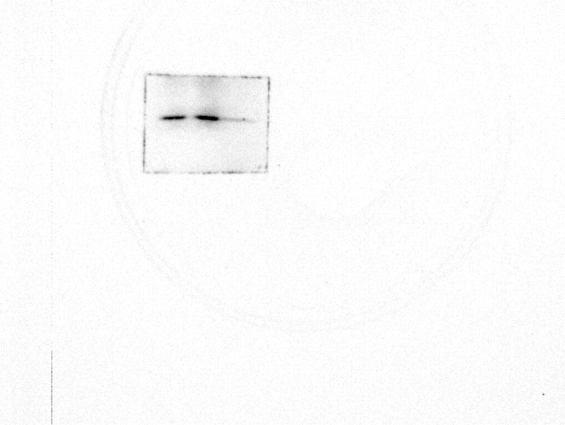 |
| 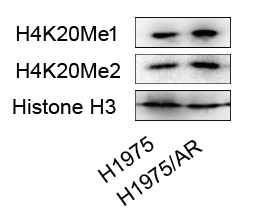  Figure 7A H3 | 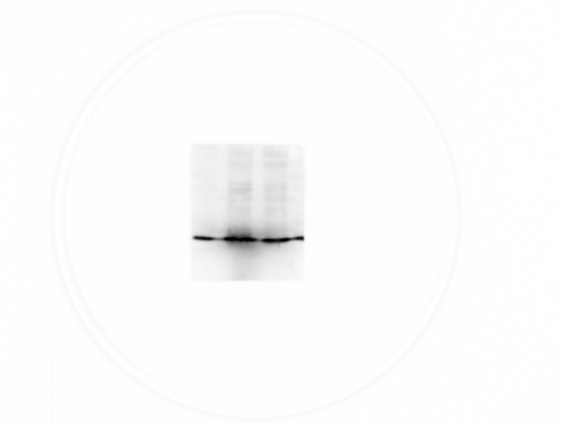 |
| 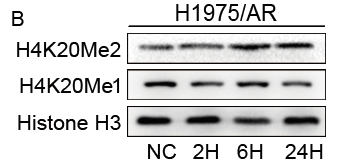  Figure 7B H4K20Me1 | 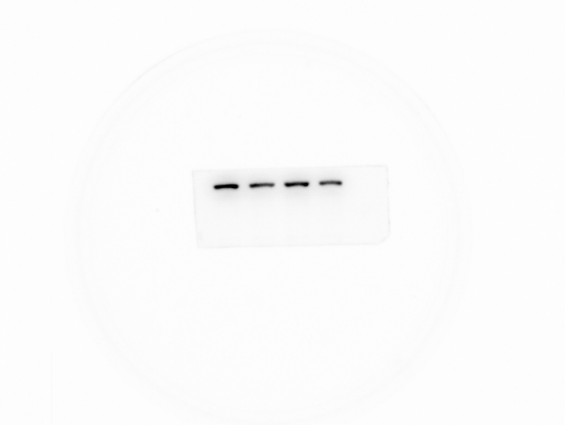 |
| 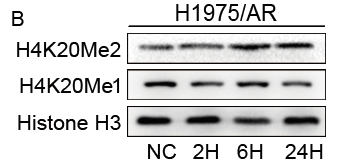  Figure 7B H4K20Me2 | 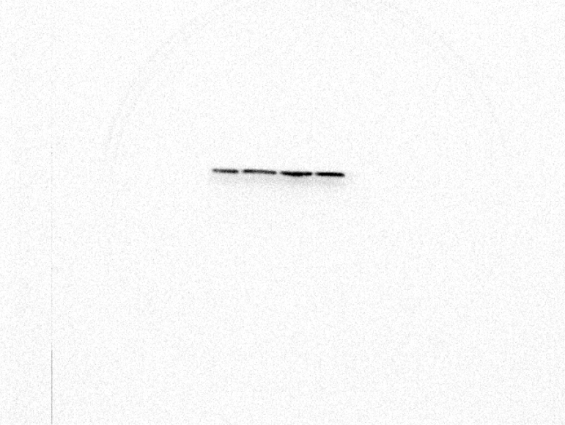 |
| 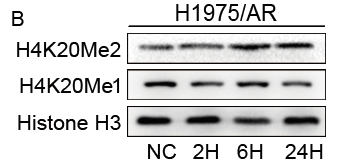  Figure 7B H3 | 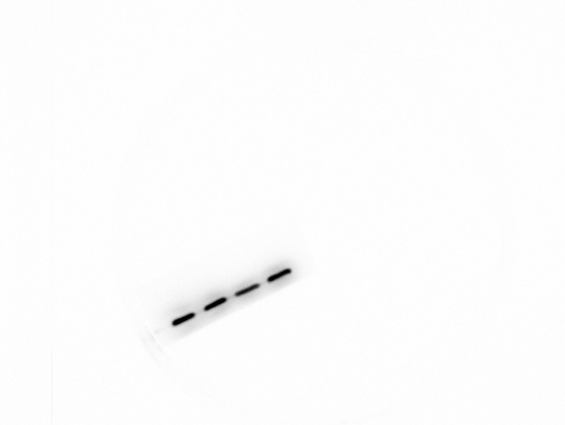 |
| 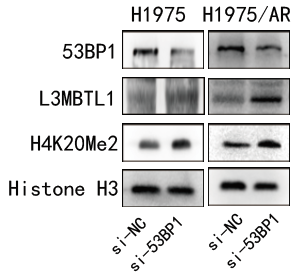  Figure 7E H1975 53BP1 | 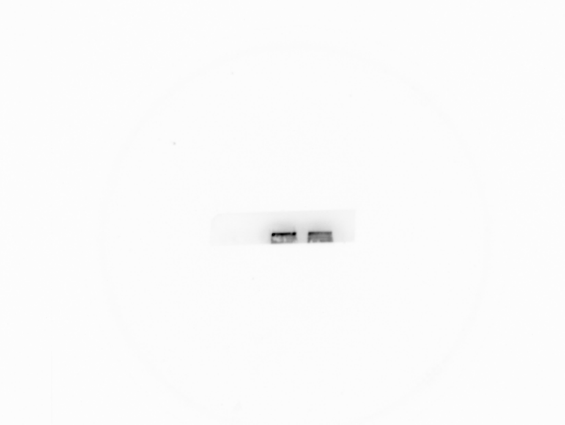 |
| Figure 7E H1975 L3MBTL1 |  |
| Figure 7E H1975 H4K20Me2 |  |
| Figure 7E H1975 H3 |  |
| Figure 7E H1975/AR 53BP1 |  |
| Figure 7E H1975/AR L3MBTL1 |  |
| Figure 7E H1975/AR H4K20Me2 |  |
| Figure 7E H1975/AR H3 |  |

| FigureS1C H1975 P-EGFR |  |
| --- | --- |
| FigureS1C H1975 EGFR |  |
| FigureS1C H1975 P-AKT |  |
| FigureS1C H1975 AKT |  |
| FigureS1C H1975 P-MAPK |  |
| FigureS1C H1975 MAPK |  |
| FigureS1C H1975 GAPDH |  |
| FigureS1C H1975/AR P-EGFR |  |
| FigureS1C H1975/AR EGFR |  |
| FigureS1C H1975/AR P-AKT |  |
| FigureS1C H1975/AR AKT |  |
| FigureS1C H1975/AR P-MAPK |  |
| FigureS1C H1975/AR MAPK |  |
| FigureS1C H1975/AR GAPDH |  |
| Figure S1F PC9&OR BRCA1 |  |
| Figure S1F PC9&OR KU80 |  |
| Figure S1F PC9&ORKU70 |  |
| Figure S1F PC9&OR RAD51 |  |
| Figure S1F PC9&OR GAPDH |  |
| Figure S2A H1975 L3MBTL1 |  |
| Figure S2A H1975/AR L3MBTL1 |  |
| Figure S2A H1975 GAPDH |  |
| Figure S2A H1975/AR GAPDH |  |
| Figure S2D PC9/OR 53BP1 |  |
| Figure S2D PC9/OR L3MBTL1 |  |
| Figure S2D PC9/OR γ-H2AX |  |
| Figure S2D PC9/OR H4K20ME2 |  |
| Figure S2D PC9/OR GAPDH |  |
| Figure S2F H1975/AR KU80 |  |
| Figure S2F H1975/AR KU70 |  |
| Figure S2F H1975/AR GAPDH |  |
| Figure S2G H1975/AR L3MBTL1 |  |
| Figure S2G H1975/AR GAPDH |  |
